# Establishment of a novel human amniotic epithelial-derived cell line, HAT, for high-yield AAV vector production

**DOI:** 10.1016/j.omtm.2025.101594

**Published:** 2025-09-12

**Authors:** Yugo Hirai, Yu-Hsin Chang, Arisa Yamamoto, Ryo Asahina, Rena Moromizato, Mawo Kinoshita, Kazuko Aizawa, Manami Miyai, Michi Kubota, Takayuki Horiuchi, Kazuaki Nakamura

**Affiliations:** 1 Chitose Laboratory Corp., Kawasaki, Kanagawa 213-0012, Japan; 2 Manufacturing Technology Association of Biologics, Tokyo 104-0033, Japan; 3 Department of Pharmacology, National Research Institute for Child Health and Development, Tokyo 157-8535, Japan

**Keywords:** adeno-associated virus, AAV vector, biomanufacturing, gene therapy, human amniotic epithelial cells, host cell line, HAT cells

## Abstract

Gene therapy using adeno-associated virus (AAV) vectors has advanced remarkably in recent decades. However, efficient AAV vector production remains challenging despite extensive efforts to optimize the commonly used HEK293 cells. Here, we describe a novel host cell line tailored for AAV vector production. Human amniotic epithelial cell line for gene and cell therapy (HAT) was generated by introducing adenovirus type 5 E1 genes into primary cells isolated from placental amnion after cesarean section. HAT cells were adapted to suspension culture conditions in a serum-free, chemically defined medium and subsequently single-cell-cloned to support future industrial applications. HAT cells exhibited robust proliferation and AAV productivity. Quality assessments revealed that HAT-produced AAV2 and AAV8 vectors had overall product quality attributes comparable to those from HEK293 cells. They were characterized by efficient genome packaging and *in vitro* infectivity, with a notably higher proportion of full capsids. Furthermore, consistent yields and product quality attributes across shake flask and benchtop bioreactor production demonstrated good scalability in HAT cells. Together, these features indicate the potential of HAT cells to provide a host cell platform that will advance biopharmaceutical manufacturing.

## Graphical abstract

**Figure:**
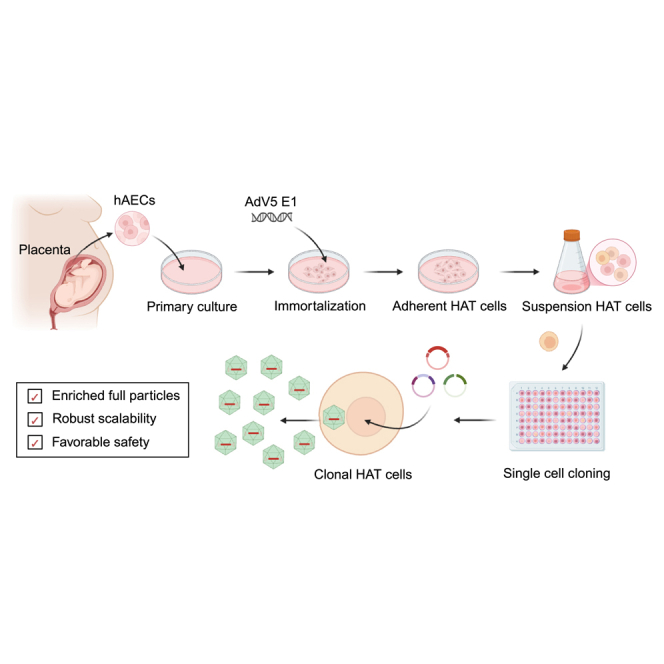

## Teaser

Hirai and colleagues established a novel cell line derived from human placental amnion for AAV production. This cell line, termed HAT, demonstrated high-titer AAV production across multiple serotypes in serum-free suspension culture conditions. HAT cells have great potential as host cells for biomanufacturing.

## Introduction

Adeno-associated virus (AAV) is a single-stranded DNA virus belonging to the *Dependoparvovirus* genus of the *Parvoviridae* family. Its 4.7-kb genome has inverted terminal repeats (ITRs) at both ends, which contain the origins of replication for viral DNA synthesis. The genome consists of two open reading frames encoding nonstructural *rep* genes and structural *cap* genes essential for viral replication and capsid assembly. With features of non-pathogenicity, sustained efficacy, and selective tissue targeting, AAV vectors have emerged as a preferred delivery vehicle for *in vivo* gene therapy. To date, eight AAV-based gene therapy products have been approved by the US Food and Drug Administration and/or the European Medicines Agency,^1^ and more than 200 clinical trials using AAV vectors are ongoing worldwide,^2^ highlighting the increasing demand for the manufacture of AAV vectors.

As suggested by the name and taxonomy classification, AAV is replication defective and relies on helper factors provided by a co-infected helper virus, such as the name-giving adenovirus (AdV), for productive propagation.^3^ To avoid potential contamination of AAV vector products with helper virus, a helper-free method based on the co-transfection of cells with three plasmids encoding essential viral and helper genes has been developed.^4^ These plasmids include an AAV *cis*-plasmid carrying the gene of interest flanked by ITRs; an AAV *trans*-plasmid encoding *rep* and *cap* genes; and a helper plasmid encoding adenoviral E2A, E4, and VARNA genes. Transfection of these three plasmids into human embryonic kidney 293 (HEK293 or 293) cells, constitutively expressing human adenovirus type 5 (AdV5) E1A and E1B genes required for AAV production, is the most common approach.^5^^,^^6^ However, AAV gene therapies typically require a high dose to achieve adequate efficacy^7^; therefore, the field requires host cells that can achieve higher product yields and improve the manufacturing processes.

HEK293 cells were originally established for basic research but are now widely used in biomanufacturing.^8^ During the past few decades, industrially relevant human cell lines, such as PER.C6, derived from human embryonic retinoblasts,^9^^,^^10^ and CAP, derived from human amniocytes,^11^^,^^12^^,^^13^ have been generated for biopharmaceutical production. Recently, a novel AAV vector production platform using CAP cells has been launched.^14^^,^^15^ Human amniotic epithelial cells (hAECs) isolated from the placental amnion represent an ideal source for generating new cell lines because they are easily accessible without additional invasive procedures and are subject to minimal ethical considerations. The pluripotent characteristics and differentiation abilities of freshly isolated hAECs have been discussed.^16^^,^^17^ Immortalized human amniotic epithelial cells, IHAECs,^18^ and iHAEs,^19^ have also been established by introducing SV40 T-antigen or HPV16 E6/E7 with hTERT into primary cells. However, the characterization of these cells has focused on their potential applications in cell therapy and regenerative medicine.

Here, we report the establishment of a novel cell line by transfecting hAECs with the AdV5 E1 gene. We designate this cell line as human amniotic epithelial cell line for gene and cell therapy (HAT). Cell line development resulted in a lead HAT clone that produced high titers across a panel of AAV vectors, with AAV5 yields up to 2.7 × 10^14^ vector genome (vg) copies per liter of culture supernatant. An increased proportion of full capsids was achieved in HAT-produced AAV2 (20.6% vs. 6.0%) and AAV8 (27.1% vs. 12.6%) vectors relative to those derived from HEK293 cells. Notably, the yield and quality of AAV vector production were preserved during scale-up of AAV8 vectors in a 3-L benchtop bioreactor. To our knowledge, this is the first application of an immortalized human placental amnion-derived cell line for viral vector production.

## Results

### Isolation and immortalization of amniotic epithelial cells

**Figure 1:**
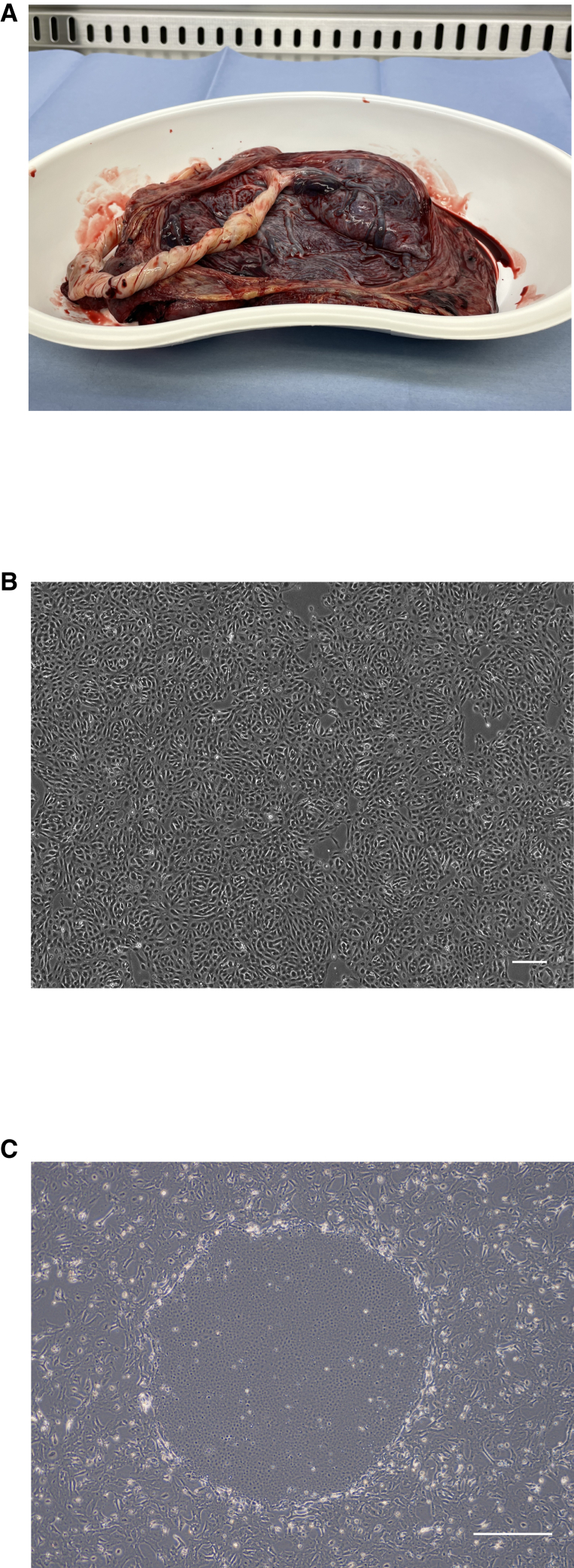
Establishment and development of novel HAT cells from placenta tissue Representative images of (A) placenta, (B) primary hAECs (scale bar: 200 μm), and (C) a transformed colony from the generation of HAT cells (scale bar: 500 μm).

Placentas were donated by healthy expectant mothers after cesarean deliveries (Figure 1A). Appropriate informed consent was obtained from all donors. hAECs were isolated from placental amnion and cultured (Figure 1B) as described in the materials and methods section. Human somatic cells have a limited lifespan; they stop multiplying and exhibit a flattened and enlarged morphology after 4–5 passages.^20^^,^^21^ We observed that hAECs derived from placentas also displayed such properties (data not shown).

Several human cell lines have been established by transformation with genes possessing AdV E1 functions.^8^^,^^9^^,^^11^ To immortalize and equip hAECs cells with constitutive E1 helper function, hAECs were transfected or electroporated with GT004, a synthetic DNA encoding AdV5 E1 genes. The transformed cells formed colonies following either transfection method (Figure 1C). These colonies were picked at approximately 30 days after E1 gene introduction and cultured continuously. Through sequential passages, some lines exhibited considerably enhanced proliferation capacity, and passaging frequency gradually increased.

Higher cell growth results in a shorter industrial cell culture period and lower manufacturing costs. Therefore, the first screen performed was based on proliferation potential. We identified several candidate cell lines that showed high growth capability and displayed the ability to reach high cell density after 7 months of uninterrupted culture (Figure S1A). In addition, constitutive expression of AdV5 E1 proteins was observed (Figure S1B). Among the resulting HAT cells, line A-2 showed high proliferation capacity (Figure S1A) and was selected for further characterization and development.

### Immortality of HAT A-2 cells

**Figure 2:**
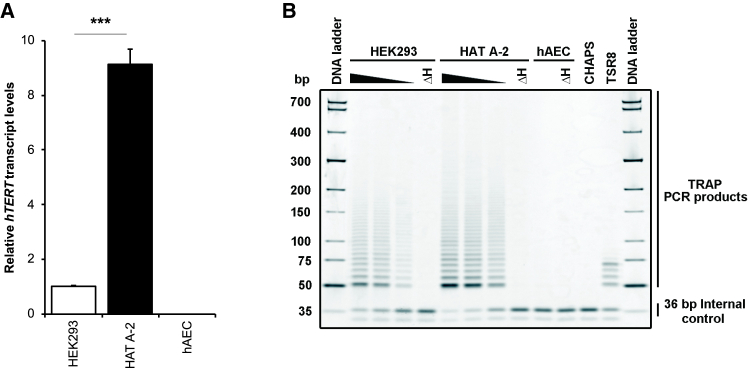
Immortality of HAT A-2 cells (A) The transcript level of *hTERT* was analyzed by RT-qPCR in triplicate samples. Relative transcript levels were normalized against levels of β-actin and then against one of the HEK293 control replicate samples using the ΔΔCt method. ∗∗∗*p* < 0.005. (B) Telomerase activity was verified using a PCR-based TRAP assay. CHAPS and TSR8, negative and positive controls, respectively; ΔH, heat inactivation control.

With the ability to be propagated continuously, we considered that HAT A-2 cells had gained immortality. To confirm the immortalization of HAT A-2 cells, we examined the expression of the immortalization marker, *hTERT*. *hTERT* transcripts were below the limit of detection in the original hAECs, consistent with previous findings.^17^^,^^22^ However, we observed a significant increase in *hTERT* transcript levels in HAT A-2 cells (Figure 2A). Overexpression of *hTERT* is accompanied by reactivation of telomerase activity in immortal cells.^23^^,^^24^ Using a telomeric repeat amplification protocol (TRAP) assay, we detected a 6-bp incremental telomerase-specific ladder in HAT A-2 cells (Figure 2B). The telomerase activity occurred in a HAT A-2 cell number-dependent manner but was absent in heat-inactivated negative control cells (ΔH) and in the original hAECs. Both *hTERT* expression and telomerase activity were higher in HAT A-2 cells than in positive control HEK293 cells, firmly indicating that HAT A-2 cells had acquired an immortal state.

### Authentication of HAT A-2 cells

A short tandem repeat analysis was conducted to authenticate the HAT A-2 cell line and to exclude cross-contamination with other human cells.^25^ The results of 10 representative markers are summarized in Table 1. HAT A-2 cells and the parental hAECs showed identical patterns, confirming the origin of HAT A-2 cells. In addition, the genetic profile of HAT A-2 cells was unique, differing from that of any reported cell line, including HEK293 cells, documented in the American Type Culture Collection (ATCC), the German Collection of Microorganisms and Cell Cultures (DSMZ), and the Japanese Collection of Research Bioresources (JCRB) Cell Bank databases. This analysis authenticated the HAT A-2 cells.

**Table 1:** Short tandem repeat profiling of hAECs and adherent HAT A-2 cells

	Amelogenin	CSF1PO 11PO	D13S317	D16S539	D21S11	D5S818	D7S820	TH01	TPOX	vWA
hAECs	X	10, 11	8, 11	9	31, 32.2	10	8, 11	7	8, 9	14, 17
HAT A-2	X	10, 11	8, 11	9	31, 32.2	10	8, 11	7	8, 9	14, 17
HEK293	X	12	12, 14	13	28, 30.2	8	11, 12	7, 9.3	11	16, 19

The alleles detected for the indicated markers are summarized. X, X homolog.

### Characterization of adherent HAT A-2 cells

**Figure 3:**
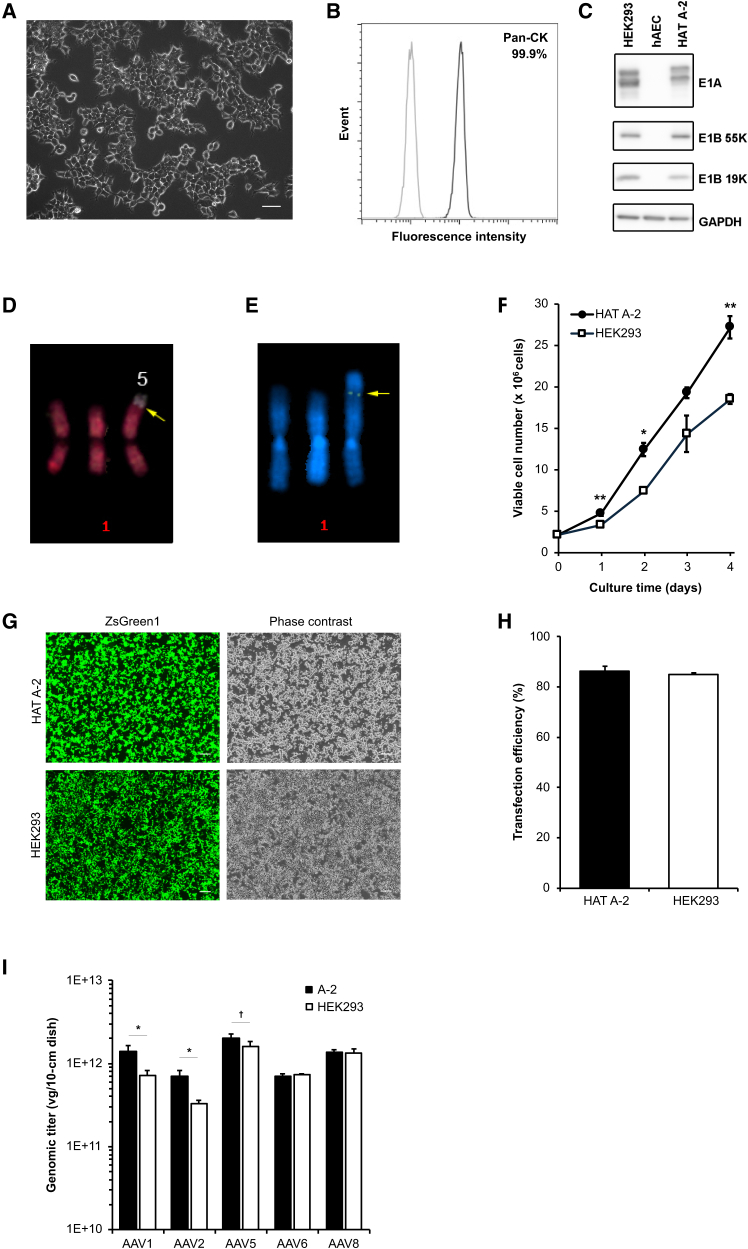
Characterization of HAT A-2 cells (A) Morphology of HAT A-2 cells under phase contrast microscopy. Scale bar: 50 μm. (B) Flow cytometric analysis of the epithelial cell marker, pan-CK. A representative flow cytometry histogram overlay plot is shown. Dark gray and light gray histograms indicate pan-CK and isotype control, respectively. (C) Protein levels of AdV5 E1A and E1B in HAT A-2 cells were examined by immunoblotting. Major E1A isoforms were detected. GAPDH served as a loading control. (D) Multicolor-FISH-based karyogram of chromosome 1 with translocated chromosome 5 (indicated by the arrow) in HAT A-2 cells. 1, chromosome 1; 5, chromosome 5. (E) FISH analysis with AdV5 E1-specific DNA probes (indicated by the arrow) of HAT A-2 cells counterstained with DAPI. 1, chromosome 1. (F) Cells were seeded at a density of 2.2 × 10^6^ cells in 10-cm dishes in triplicate. Cell number was counted daily for 4 days using a Vi-CELL XR analyzer (Beckman Coulter). ∗*p* < 0.05 and ∗∗*p* < 0.01. (G) Cells were transfected with a vector expressing the green fluorescent protein ZsGreen1. ZsGreen1 expression was examined under fluorescence microscopy at 48 h post-transfection. Scale bar: 200 μm. (H) Percentage of ZsGreen1-positive cells at 48 h post-transfection was quantified by flow cytometry. (I) AAV vectors were produced by a triple plasmid transfection method. Three (for AAV1, AAV6, and AAV8) or four (for AAV2 and AAV5) independent experiments were performed, and genomic titers were measured by ddPCR. ∗*p* < 0.05 and †*p* = 0.057.

HAT A-2 cells displayed a typical epithelial-like cobblestone morphology with good adherence to the culture plate (Figure 3A). They were able to grow at high density without apparent contact inhibition (Figure 3G). The great majority of the HAT A-2 cells (99.9%) were positive for the epithelial cell marker, pan-cytokeratin (pan-CK),^26^ confirming the epithelial origin of the cells (Figure 3B). HAT A-2 cells constitutively expressed AdV5 E1 proteins (Figure 3C). The levels of E1A, E1B 19K, and E1B 55K were comparable with those in HEK293 cells. To elucidate whether E1 sequences are stably integrated into a chromosome, fluorescence *in situ* hybridization (FISH) and multicolor FISH were performed using an AdV5 E1 DNA probe to map the physical location of E1 genes. All examined cells (*n* = 10) exhibited chromosome 1 trisomy. Translocations between one copy of the trisomic chromosome 1 and chromosome 5 [t(1;5)] (Figure 3D) and insertion of transgenic E1 (indicated by an arrow) within the translocation site between chromosomes 1 and 5 (Figure 3E) were visualized in all examined cells, indicating that HAT A-2 cells were probably derived from a single cell with the aforementioned genetic characteristics.

To determine whether E1 integration exhibits a site preference in the hAECs, we analyzed the integration site in HAT A-12, which is another immortalized line capable of reaching high cell density (Figure S1A). FISH signals were consistently detected at the telomeric region of the long arm of chromosome 17 in all examined cells (*n* = 10) (Figure S2A). In three out of the ten cells, the telomeric region appeared to be translocated to chromosome 10 (Figure S2B). The distinct E1 integration patterns observed among HAT A-2 and A-12 cells indicate that the high-proliferation phenotype may not be solely determined by a specific E1 insertion site.

To assess long-term growth stability, HAT A-2 cells were subcultured every 3 to 4 days, and viable cell numbers were recorded at each passage over 40 passages (Figure S3). For data comparability, only the results from day 3 passages are shown. A stable proliferation pattern was observed, indicating that proliferation capacity was not affected by passage number. To further evaluate proliferation characteristics, a head-to-head comparison between HAT A-2 and HEK293 cells was performed (Figure 3F). HAT A-2 cells consistently showed higher proliferation, with significant differences observed at multiple time points during the 4-day culture period.

For AAV vector production, a candidate host cell is required to have high transfection efficiency because triple transfection is currently the most established method. To examine transfection efficiency, cells were transfected with pAAV-ZsGreen1, a vector expressing a green fluorescent reporter protein, using a cationic lipid-mediated reagent. ZsGreen1 expression was confirmed using fluorescence microscopy (Figure 3G) and flow cytometry (Figure 3H). HAT A-2 cells showed excellent transfection efficiency (86.4%), comparable with that of HEK293 cells (85.1%). These features indicate that HAT A-2 cells are suitable as a host cell platform.

### Production of AAV vectors by adherent HAT A-2 cells

Next, we assessed the capability of HAT A-2 cells to produce recombinant AAV vectors using a triple plasmid transfection method and the ZsGreen1 reporter as a gene of interest. AAV titers of HAT A-2 cells were comparable with those of HEK293 cells across the five serotypes tested, with significant increases observed for AAV1 (approximately 2.0-fold) and AAV2 (approximately 2.1-fold) in HAT A-2 cells (Figure 3I). The higher proliferation potential of HAT A-2 cells compared with HEK293 cells (Figure 3F) prompted us to examine separate experimental batches of AAV1 and AAV2, which had shown significantly increased titers in HAT A-2 cells, to determine whether the increased volumetric productivity observed for AAV1 and AAV2 resulted from enhanced cell-specific productivity or merely higher cell numbers. To address this, cell numbers prior to harvest were counted, and the titers were normalized against cell number to calculate cell-specific productivity.

Under the triple plasmid transfection conditions, the growth difference between the two cell lines narrowed, with HAT A-2 cells showing an approximately 1.2-fold increase in cell number compared with HEK293 cells (Figure S4A). This indicates that the higher volumetric yields from HAT A-2 cells (Figure S4B) reflected improved cell-specific productivity rather than elevated cell density. Per-cell AAV production was increased by approximately 1.6-fold for AAV1 and 1.8-fold for AAV2 in HAT A-2 cells compared with that in HEK293 cells (Figure S4C).

### Suspension adaptation of HAT A-2 cells

Adherent cell-based approaches are widely used for industrial-scale manufacturing; however, lengthy and cumbersome cell expansion processes make large-scale manufacturing challenging using the adherent platform. In recent years, the industry has implemented serum-free suspension-adapted cell-based processes to enhance safety and lower costs.^27^^,^^28^^,^^29^ Accordingly, HAT A-2 cells were adapted to serum-free, chemically defined shake flask suspension culture. Adherent HAT A-2 cells were first cultured in HE100, a chemically defined, serum-free medium. They were subsequently transferred to serum-free suspension culture via stepwise adaptation using HE200, HE300AZ, and HE400AZ, which have progressively increasing nutrient levels to support higher viable cell density. In parallel, HAT A-2 cells were adapted to several other media widely used for biomanufacturing. Cell growth was assessed in HE400AZ, Expi293 Expression Medium (Expi293), and viral production medium (Figure S5A). HAT A-2 cells proliferated robustly in all three media. A significantly higher viable cell density of 14.1 × 10^6^ cells/mL was achieved at day 6 in HE400AZ compared with the densities achieved in the other media. Based on this result, HE400AZ was selected as the standard medium for subsequent development.

Next, transfection reagents compatible with suspension AAV production were tested. AAV2 and AAV8 vectors were produced by triple plasmid transfection using three commonly used reagents and their recommended protocols (Figure S5B). Plasmid DNA was transfected at a 1:1:1 ratio. All conditions supported AAV vector production, with FectoVIR-AAV consistently yielding the highest titers. Further modification of the FectoVIR-AAV protocol by increasing complexation volume and incubation time resulted in a 10%–20% improvement in yield. Accordingly, this modified protocol was selected for subsequent experiments.

### Single-cell cloning of HAT A-2 cells

To align with standard industry practices for ensuring batch-to-batch consistency, suspension-adapted HAT A-2 cells were subjected to single-cell cloning using an automated sorting instrument. A series of screening processes was performed to identify clones with robust proliferation and superior AAV productivity. Results from ten representative clones demonstrated the range of observed characteristics. From more than 900 single-cell clones analyzed (data not shown), a lead clone, HAT A2C1, was selected that exhibited enhanced proliferation (Figure S6A) and an approximately 2.3-fold greater volumetric AAV2 productivity compared with the parental HAT A-2 suspension cells (Figure S6B).

### Characterization of clone HAT A2C1

**Figure 4:**
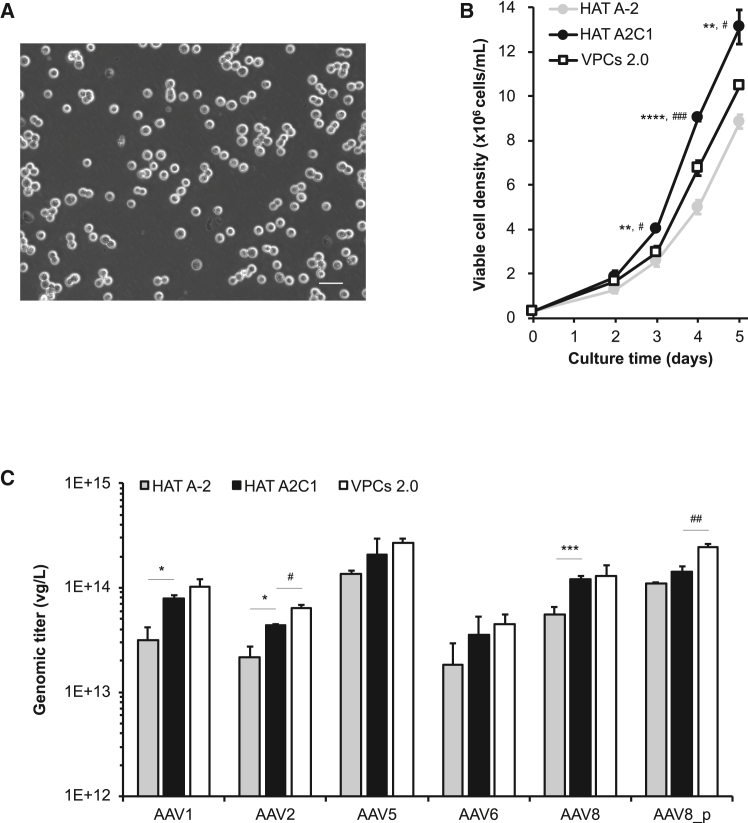
Evaluation of clonal HAT A2C1 suspension cells (A) Morphology of HAT A2C1 cells under phase contrast microscopy. Scale bar: 50 μm. (B) HAT A2C1, HAT A-2, and VPCs 2.0 cells were seeded at a density of 0.3 × 10^6^ cells/mL in a 125-mL shake flask containing 30 mL medium in triplicate. Cell numbers were measured daily for 5 days. ∗∗*p* < 0.01 and ∗∗∗∗*p* < 0.001 vs. HAT A-2 cells; #*p* < 0.05 and ###*p* < 0.005 vs. VPCs 2.0 cells. (C) AAV vectors were produced using the standard triple plasmid transfection method. AAV8 vectors were produced under two experimental conditions to assess the impact of different helper plasmids. Genomic titers were measured by ddPCR.∗*p* < 0.05 and ∗∗∗*p* < 0.005 vs. HAT A-2 cells; #*p* < 0.05 and ##*p* < 0.01 vs. VPCs 2.0 cells.

The clonal HAT A2C1 cells grew as a single-cell suspension. No obvious clumps or cell debris were observed under microscopy examination during routine cell maintenance (Figure 4A). HAT A2C1 cells; the parental HAT A-2 cells; and VPCs 2.0 cells, a HEK293F-derived clonal cell line optimized for AAV vector production, were initially characterized in parallel. HAT A2C1 cells demonstrated significantly higher growth than both HAT A-2 and VPCs 2.0 cells, reaching a viable cell density of 13.1 × 10^6^ cells/mL on day 5 (Figure 4B).

AAV productivity was then evaluated. Among the five tested serotypes, HAT A2C1 cells consistently produced higher titers than parental HAT A-2 cells, ranging from 3.5 × 10^13^ vg/L to 2.1 × 10^14^ vg/L (Figure 4C). Notably, despite the culture medium and transfection reagent being optimized for the HEK293-based system, HAT A2C1 cells exhibited comparable AAV productivity to VPCs 2.0 cells in four of the five serotypes. These results support HAT A2C1 as a robust and high-performing host cell line for AAV vector production.

To further examine the production potential of HAT A2C1 cells, we adjusted the transfection parameters by varying the plasmid ratio for AAV2. Among various conditions tested, one modified ratio led to an approximately 3.2-fold increase in AAV2 titer compared with the standard 1:1:1 conditions (Figure S7). In addition, increases of 1.1-fold in AAV1, 1.3-fold in AAV5, and 1.7-fold in AA6 were also observed. Although this experiment was not performed in direct parallel with the standard 1:1:1 conditions, the improvement indicates the feasibility for further optimization. We also evaluated an alternative pPLUS-AAV-helper plasmid for AAV8 production (AAV8_p) (Figure 4C). The change of helper plasmid alone led to a modest increase (approximately 20%) in vector yield. The full-to-empty capsid ratio, an essential quality attribute, also showed an upward trend under these conditions (Figure S8). Together, these observations indicate that refinement of transfection parameters can enhance both yield and quality of AAV vectors, highlighting the potential of HAT A2C1 cells.

### Characterization of HAT A2C1-produced AAV2 vector

**Figure 5:**
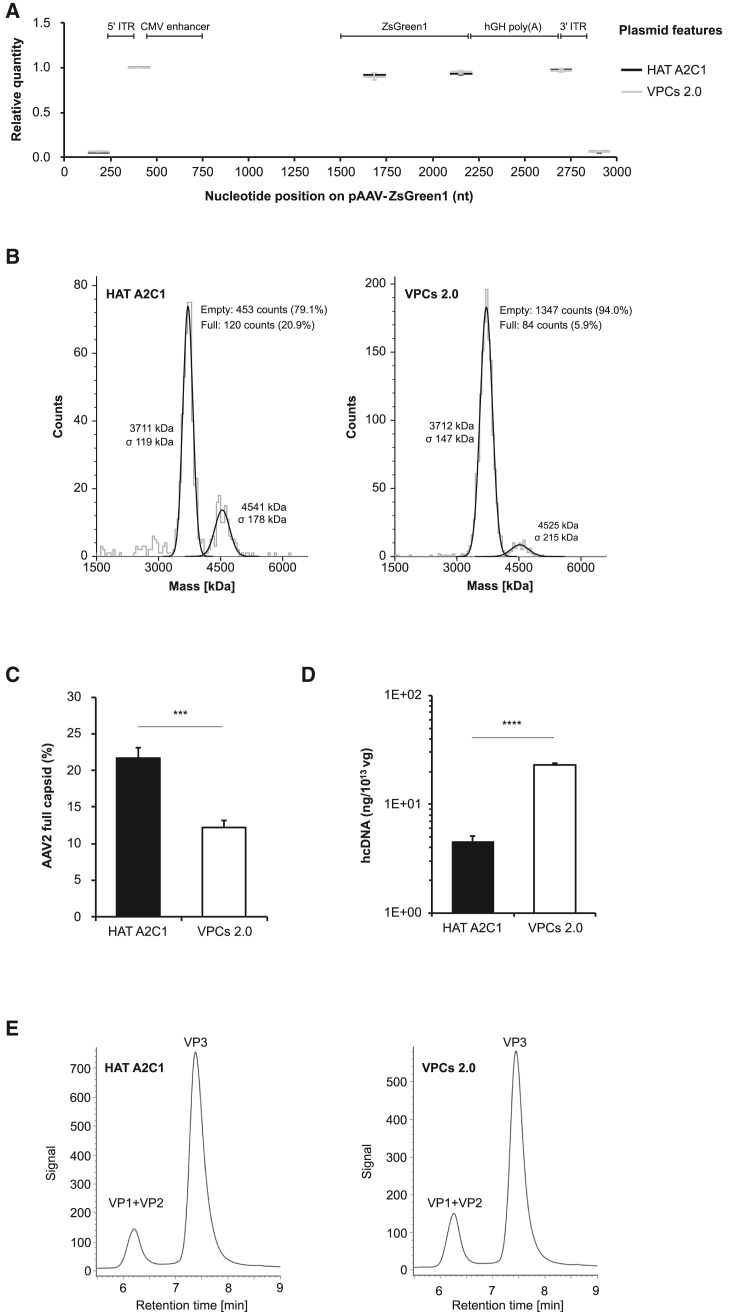
Characterization of HAT A2C1-produced AAV2 vectors (A) Genome regions packaged in AAV2 particles were analyzed by ddPCR using six primer/probe sets targeting various regions of the AAV genome, including ITR, ZsGreen1 transgene, and vector backbone residuals. The relative quantity of each target region was calculated by normalizing the raw ddPCR concentration value (copies/μL) against the “5′ ITR to CMV enhancer” region. The normalized values were plotted as horizontal bars spanning the nucleotide positions of the targeted region on the plasmid (*x* axis). Normalization was performed for each replicate prior to averaging across three independent experiments. The positions of functional elements in pAAV-ZsGreen1 are shown to indicate the location of each ddPCR target region. (B) Capsid contents of affinity-purified AAV2 vectors were analyzed by mass photometry. Representative mass histograms are shown. (C) Genomic and capsid titers of crude AAV2 lysates were measured using a duplex ddPCR-based kit. The percentage of full capsids was calculated as the ratio of genome to capsid titers. ∗∗∗*p* < 0.005. (D) Residual hcDNA in affinity-purified samples was quantified using a qPCR-based kit specific to human DNA. ∗∗∗∗*p* < 0.001. (E) Chromatographic separation of viral proteins of AAV2 vectors using an LC-FLR/MS-based method. Co-elution of VP1 and VP2 was observed.

To evaluate the quality of HAT A2C1-produced AAV2 vector, we first examined the packaged vector fragment because incomplete or partial genome packaging may result in suboptimal potency. Simplex droplet digital PCR (ddPCR) with six primer/probe sets (Table S1) targeting different regions spanning from the plasmid backbone through the AAV genome was performed (Figure 5A). The resulting profiles were comparable with those from VPCs 2.0 cells, with near-equivalent levels detected from the 5′ ITRs through the ZsGreen1 gene to the 3′ ITRs. These results provide evidence of consistent packaging efficiency across the entire AAV genome.

Next, the proportions of full and empty capsids were measured using mass photometry (Figure 5B). The populations of empty and full capsids were clearly resolved, with the empty peaks detected close to the expected molecular weight. A 3.4-fold higher proportion of full capsids was observed for AAV2 vectors produced by HAT A2C1 cells (20.6%) compared with those from VPCs 2.0 cells (6.0%) in three biological replicates. This finding was further supported by a 1.8-fold increase in the genome-to-capsid ratio observed for HAT A2C1-produced AAV2 (21.7%) relative to that from VPCs 2.0 cells (12.3%), as determined by duplex ddPCR simultaneously measuring genome and capsid titers (Figure 5C).

Following confirmation of efficient genome packaging and a high percentage of full AAV capsids, the levels of residual host cell DNA (hcDNA), a process-related impurity in AAV gene therapy products, in affinity-purified samples were quantified using a commercial human-specific qPCR-based kit (Figure 5D). To confirm whether HAT DNA can be reliably detected by the kit, the concentration of genomic DNA from whole-cell lysates of both cell lines was quantified using the qPCR-based kit and normalized against that determined by NanoDrop spectrophotometry (Figure S9). The resulting ratios showed comparable values between the two cell lines, indicating that the kit provides consistent quantification without substantial bias under equivalent DNA input. The concentration of residual hcDNA in HAT A2C1-produced AAV2 vectors (4.5 ng/10^13^ vg) was significantly lower than that in the AAV2 vectors produced from VPCs 2.0 cells (22.9 ng/10^13^ vg). These results indicate that the reduced hcDNA levels observed in HAT A2C1-derived vectors are unlikely to result from inefficient detection by the kit but rather reflect lower levels of hcDNA impurities.

Another critical quality attribute of AAV vectors, the viral capsid protein (VP) ratio, was examined using liquid chromatography (LC)-fluorescence (FLR)/mass spectrometry (MS) analysis of affinity-purified AAV2 samples. All VPs were detected with masses matching their theoretical values, confirming the accuracy of the spectral identification (Table S2).^30^ Comparable levels of VP1+VP2 and VP3 proteins, with VP1+VP2:VP3 ratios closely aligned to the ideal 1:5 (1:5.5 for HAT A2C1 vs. 1:4.4 in VPCs 2.0), were observed in both HAT A2C1 and VPCs 2.0 samples (Figure 5E). Together, these results indicate the favorable characteristics of AAV2 vectors produced by HAT A2C1 cells.

### Infectivity of HAT A2C1-produced AAV2 vectors

**Figure 6:**
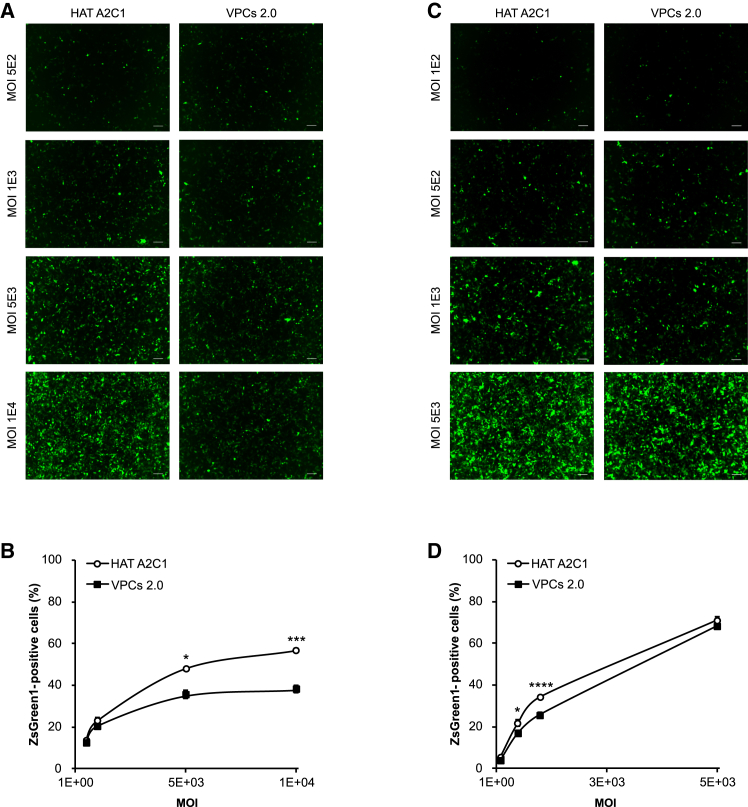
Transduction of HAT A2C1-produced AAV2 vectors (A) Transduction of HEK293 cells with affinity-purified AAV2-ZsGreen1 produced from HAT A2C1 and VPCs 2.0 cells at the indicated MOIs. The expression of ZsGreen1 was examined under fluorescence microscopy at 4 days post-transduction. Scale bar: 100 μm. (B) The percentage of ZsGreen1-positive cells was measured using a Countess 3 FL automated counter. The results were calculated from biological triplicates. (C) Transduction of HEK293 cells with full particle-enriched samples from AEX purification at the indicated MOIs. The expression of ZsGreen1 was examined under fluorescence microscopy at 4 days post-transduction. Scale bar: 100 μm. (D) The percentage of ZsGreen1-positive cells was measured using a Countess 3 FL automated cell counter. A single transduction was performed, and error bars were generated from technical triplicates.

Next, the potency of HAT A2C1-produced AAV2 vectors was evaluated using an *in vitro* transduction assay in HEK293 cells with affinity-purified samples at MOIs ranging from 5 × 10^2^ to 1 × 10^4^ vg/cell (Figure 6A). AAV2 vectors produced from both cells exhibited potent transduction across all MOIs tested. However, at equivalent vector input, HAT A2C1-produced AAV vectors reproducibly yielded significantly higher numbers of ZsGreen1-positive cells compared with those from VPCs 2.0 cells in three biological replicates (Figure 6B). Given the marked difference in empty-to-full ratios between the two preparations, the outcomes may simply reflect the larger proportion of empty capsids in the VPCs 2.0 samples, which could dilute infectivity (Figures 5B and 5C).

To directly assess the intrinsic transduction potency, we prepared an additional batch of AAV2 samples enriched for full particles through two consecutive rounds of anion exchange (AEX) chromatography. After the AEX purification process, the proportion of full particles became comparable between the two preparations (84.4% for HAT A2C1 vs. 77.9% for VPCs 2.0) (Figure S10). Both AEX vector preparations exhibited enhanced transduction potency, reaching approximately 70% ZsGreen1-positive cells at an MOI of 5 × 10^3^ vg/cells, indicating that the removal of empty capsids and/or impurities improved overall infectivity (Figures 6C and 6D). Under these more comparable conditions, HAT A2C1-produced vectors continued to show an observable trend of higher transduction efficiency compared with the VPCs 2.0 vectors (Figure 6D). The remaining modest difference can be attributable to several factors, including the slightly higher proportions of full particle in the HAT A2C1 preparations.

### Evaluation of HAT A2C1 cell scalability for AAV8 vector production

**Figure 7:**
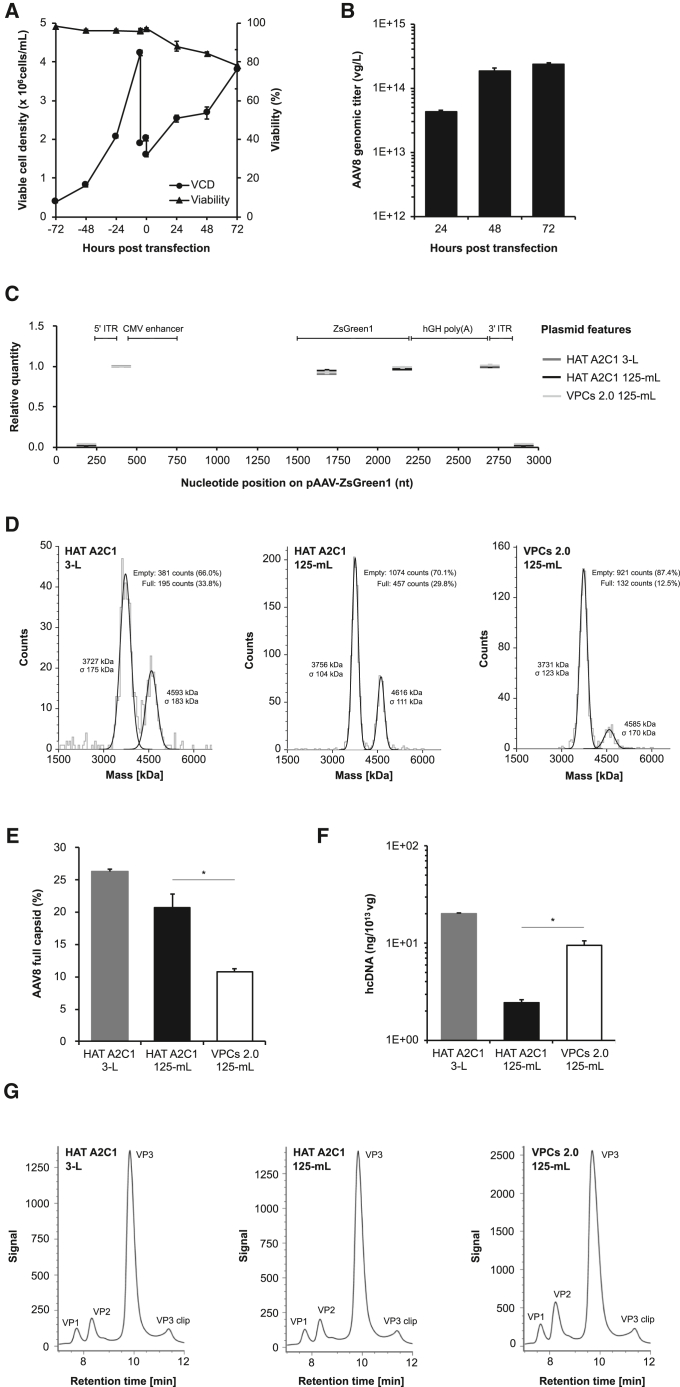
Assessment of HAT A2C1 cell scalability for AAV8 vector production (A) Large-scale cultivation and AAV8 vector production were performed in a 3-L single-use orbital shaken bioreactor. Cell growth and cell viability were monitored from inoculation to AAV harvest. Triple plasmid transfection was initiated at time 0 using the pPLUS AAV-Helper plasmid. Error bars are generated from technical replicates collected from a single batch run to account for sampling variability. VCD, viable cell density. (B) AAV8 vectors were harvested at 24, 48, and 72 h post-transfection, and genomic titers were measured by ddPCR. Error bars are generated from technical replicates from a single batch run. (C) Packaged genome regions in AAV8 particles were analyzed by ddPCR using six primer/probe sets covering different regions of the AAV genome, including ITR, ZsGreen1 transgene, and vector backbone residuals. Relative quantity of each target region was calculated by normalizing the raw ddPCR concentration value (copies/μL) against the “5′ ITR to CMV enhancer” region. The normalized values were plotted as horizontal bars spanning the nucleotide positions of the targeted region on the plasmid (*x* axis). The relative positions of functional elements on pAAV-ZsGreen1 are shown to indicate the location of each ddPCR target region. (D) Capsid contents of affinity-purified AAV8 vectors were analyzed by mass photometry. Representative mass histograms are shown. (E) Genomic and capsid titers of crude AAV8 lysate were measured using a duplex ddPCR-based kit. The percentage of full capsids was calculated as the ratio of genome to capsid titers. ∗*p* < 0.05. (F) Residual hcDNA in affinity-purified samples was quantified using a qPCR-based kit specific to human DNA. ∗*p* < 0.05. (G) Chromatographic separation of AAV8 vector viral proteins using an LC-FLR/MS-based method. For qualitative comparison, data from the bioreactor run are presented with data from shake flask runs under the same experimental conditions. Three independent experiments were conducted for the shake flask conditions, with error bars generated from biological triplicates. For the bioreactor conditions, a single batch was analyzed, and error bars were generated from technical triplicates. For quality assessment, bioreactor samples were collected at 72 h post-transfection.

To meet the growing need for AAV gene therapy, the industry is shifting toward large-scale manufacturing in bioreactors. To assess the scalability of HAT A2C1 cells and to examine whether the observed trends in HAT A2C1-produced AAV2 vectors also apply to other serotypes, we performed a scale-up of AAV8 vector production in a 3-L single-use bioreactor. Based on the relatively higher titers and full particle ratios produced in prior experiments under AAV8_p conditions (Figures 4C and S8), pPLUS-helper plasmid was chosen for large-scale AAV8 production. Robust cell growth was observed, with high viability maintained from inoculation throughout the production process (Figure 7A). AAV8 titers increased steadily over the production period. Notably, the bioreactor yield at 48 h post-transfection (1.8 × 10^14^ vg/L, Figure 7B) had already exceeded that from flask-scale production at 72 h (1.4 × 10^14^ vg/L, Figure 4C).

The quality attributes of AAV8 vectors were then assessed using the same panel of analytical assays described in Figure 5, to see whether the quality was preserved during scale-up. Side-by-side comparisons were conducted between vectors produced in shake flasks and in the 3-L bioreactor. The genome profiles of packaged vectors (Figure 7C) and capsid protein composition (Figure 7G; Table S3) were comparable between the two production scales, indicating consistent genome packaging and VP composition at scalable production. Notably, there was a tendency for the proportion of full capsids in the bioreactor-produced vectors to be higher compared with that from shake flask-produced vectors (Figures 7D and 7E). Consistent with the findings from AAV2 vectors, HAT A2C1-produced AAV8 vectors exhibited a higher proportion of full capsids (27.1% vs. 12.6%) and lower residual hcDNA levels (2.4 vs. 9.5 ng/10^13^ vg) (HAT A2C1 125-mL vs. VPCs 2.0 125-mL, Figure 7F) compared with those from VPCs 2.0 cells in three biological replicates.

## Discussion

After several decades of development, AAV vector-mediated gene therapy is now an option for treating retinal dystrophy, spinal muscular atrophy, Duchenne muscular dystrophy, aromatic L-amino acid decarboxylase deficiency, and hemophilia.^31^ These exciting breakthroughs for rare monogenic diseases have encouraged the field to meet the challenges of developing gene therapies for prevalent, multigenic diseases. AAV vector-based gene therapies for various diseases, including cardiac dysfunctions, metabolic diseases, central nervous system diseases, and cancers are currently undergoing clinical trials.^31^ In response to the increasing demand for AAV vectors, more cost-effective and scalable solutions for AAV vector manufacture are being sought. However, manufacturing capacity cannot keep up with clinical progress, resulting in extraordinarily high prices and limited availability of AAV vector-based drugs for patients. One of the most adopted approaches to improving AAV vector production yield has focused on developing host cell lines and optimizing upstream production processes. Despite extensive subcloning of HEK293 cells, modern serum-free suspension culture in refined media, high-performance transfection reagents, and design-of-experiment-optimized transfection protocols, the maximum crude harvest titers obtained from HEK293-based systems are between 1 × 10^14^ and 1 × 10^15^ vg/L,^32^^,^^33^ which is insufficient to meet clinical needs, particularly for systemic indications. For example, Elevidys, a gene therapy for Duchenne muscular dystrophy, uses a dose of 1.33 × 10^14^ vg/kg^34^; for a 4-year-old boy weighing approximately 16 kg, this corresponds to a total dose of approximately 2.1 × 10^15^ vg per patient. At a production titer of 1 × 10^14^ vg/L with assuming 20% downstream yield, AAV vectors produced per 200-L bioreactor run would only produce enough vector for about two doses.^7^^,^^35^ Considering the estimated incidence of DMD at 1 in 3,500 live births,^36^ the current productivity benchmarks may pose challenges for meeting supply demands.

When Frank Graham attempted to transform human embryonic kidney cells in 1973, only one transformed colony survived.^8^ The cells of this single colony established the original HEK293 cell line from which all HEK293 progeny cell lines in use today are derived. A recent systematic study elucidated the genomic and transcriptomic variations among six HEK293 cell lines.^37^ Although distant from the original HEK293 cells, suspension derivatives (293-F, Freestyle 293-F, and 293-H cells) showed similar gene expression profiles. Interestingly, 293-F and 293-H cells are independent clonal isolates selected from parental HEK293 cells, but the comparably close clustering of 293-F and 293-H cells indicates very similar traits between these progeny cell lines. Based on these findings, we decided to increase the diversity and potency of the host cells for AAV vector manufacture by establishing a new tailored cell line instead of developing another clone from existing HEK293 cells. However, using human embryonic kidney tissue to generate a cell line is ethically inappropriate and impractical today. It is also suboptimal because our aim is to develop a diversified host cell. Instead, we chose hAECs as the initial cell type because they have several advantages. First, the risk of exposure to infectious agents is relatively low in fetal tissues, including the placenta. The negative results for HIV, HBV, HCV, HTLV-1, and *Treponema pallidum* in the recruited expectant mothers supported the safety of the raw materials used in this study. In addition, non-tumorigenic properties and a lack of ethical issues distinguish hAECs from other fetal-related cell types.^22^^,^^38^^,^^39^ Importantly, placentas can be obtained without additional invasive procedures following cesarean deliveries. Therefore, hAECs are abundant and easy to collect, and it is possible to prepare a large population for screening promising candidates in the early stage of cell line establishment.

During 2 years of recruitment, 20 expectant mothers enrolled in this study and donated placental tissues with informed consent. Over 500 transformed colonies were picked for the bulk starting pool. After screening, 10 promising candidates with accelerated growth potential were selected and named HAT cells. Adherent HAT A-2 cells were demonstrated to have high proliferative capacity, transfection efficiency, and AAV vector production capacity, equivalent to or greater than those of HEK293 cells. It is interesting to note that HAT A-2 cells are trisomic for chromosome 1 and that the E1 transgene used to immortalize hAECs is inserted in the [t(1;5)] translocation site, while the E1 integration site is located in the telomere of the long arm of chromosome 17 in HAT A-12 cells. Although the expression levels of E1A and E1B are similar in HAT A-2 and HEK293 cells, analysis of differential gene expression patterns associated with this distinct chromosomal region could enable the identification of specific target genes and cellular pathways that are involved in the functional consequences in HAT A-2 cells.

Suspension culture provides significantly improved scalability for industrial mammalian cell culture, allowing large-scale production in bioreactors with capacities of thousands of liters. We therefore adapted HAT A-2 cells to grow in suspension culture. HAT cells demonstrated broad compatibility with various culture media and transfection reagents and supported efficient AAV vector production across multiple serotypes, indicating their flexibility for process development and applicability to scalable biomanufacturing. To confirm scalability, we examined the performance of the top A2C1 single-cell clone in a 3-L benchtop bioreactor. Cell proliferation and AAV productivity were highly comparable during the scale-up. The experimental constraints and operational complexity of bioreactor production mean that it is difficult to strictly replicate identical conditions at the early stage of development to generate biological replicates. Additional runs of bioreactor production were conducted under slightly varied conditions and are therefore not included in Figure 7 for direct comparison. Nevertheless, similar trends were observed among the bioreactor runs.

Safety is the top priority in biomanufacturing; therefore, HAT A2C1 cells were subjected to a comprehensive safety assessment by an independent third-party laboratory using a research cell bank, in accordance with the International Council for Harmonisation of Technical Requirements for Pharmaceuticals for Human Use Q5A guidelines (Table S4). HAT A2C1 cells passed sterility testing. Moreover, no adventitious agents, extraneous agents, viral contaminants, viral DNA, retroviral reverse transcriptase, or mycoplasma were detected. In parallel with safety testing, identity testing, including short tandem repeat profiling, was conducted and found to be consistent between HAT A2C1 cells and the parental HAT A-2 adherent cells. Together, these results support the safety and the genetic identity of HAT A2C1 cells.

In addition to evaluating the safety of the host cell line, we also assessed the quality of AAV vectors produced by HAT A2C1 cells. As part of the evaluation, packaging efficiency was examined to reduce the potential overestimation of genomic titers from the use of ITR-specific ddPCR probes. AAV titers were quantified using six primer/probe sets targeting different regions of the vector genome. Comparable copy numbers observed across the AAV genome indicated that the packaging efficiency was nearly equivalent. Notably, ZsGreen1, the gene of interest used in this study, is a relatively small transgene compared with typical therapeutic payloads. Therefore, future evaluation using larger transgene payloads will be necessary to fully validate packaging capacity under more therapy-relevant conditions.

Product- or process-related impurities can influence the overall efficacy of a therapy and can also increase the risk of adverse events. For example, empty capsids, which lack the therapeutic gene, can reduce efficacy and elicit an immune response; however, removing excess empty capsids remains a significant challenge in the AAV downstream process.^40^ The significantly higher proportions of full capsids observed for HAT A2C1-produced AAV vectors mean that less starting material needs to be fed into downstream processing to achieve a given therapeutic dose. This reduction in the viral vector input may help improve safety margins. Although low levels of hcDNA were observed in AAV vectors produced by HAT A2C1 cells, this is a preliminary observation because residual hcDNA impurities are typically assessed in fully purified clinical grade preparations. However, our results indicate that fewer impurities are present at harvest from HAT A2C1 cells than from VPCs 2.0 cells, resulting in a reduced burden on downstream purification processes. Recently, apoptotic DNA fragmentation was reported as the major source of product-related DNA impurities in AAV vector manufacturing.^41^ Consistent with this, a modest increase in hcDNA was observed in bioreactor-produced samples compared with shake flask samples. This difference may reflect the slightly lower cell viability at harvest (bioreactor: 78.2% vs. shake flask: 93.8%, data not shown). Additional process development of bioreactor systems to maintain higher cell viability may help mitigate hcDNA levels and improve product quality. To ensure efficacy, assessment of the biological potency of viral vectors is critical. To this end, we performed *in vitro* transduction assays and confirmed that HAT A2C1-produced AAV2 vectors were functionally active. Our preliminary experiments using AEX-purified samples indicated that HAT A2C1-produced vectors exhibited potency at least comparable with that of VPCs 2.0. Further studies with fully purified samples are required to accurately characterize its functional advantages.

This study is the first to investigate the clinical applicability of immortalized cells derived from human placental tissues for viral vector production. Accordingly, off-the-shelf culture media and transfection reagents were used, which have all been designed for use with HEK293-based suspension systems. In addition, protocols have not been optimized for HAT A2C1 cells. However, AAV titers from HAT A2C1 cells were comparable with those reported from HEK293 platforms.^42^^,^^43^^,^^44^ Further improvements are expected to be achieved for HAT A2C1 cells by developing optimized media, reagents, and production processes, as suggested by preliminary tests on transfection conditions, DNA input amount, and plasmid design. In addition, our preliminary results also indicate that HAT A-2 cells, like HEK293 cells, can produce other viral vectors, including lentiviral and retroviral vectors (data not shown). Future investigation of HAT cells is needed to enable their use in diverse virus production systems and to improve virus vector production for gene therapy.

In summary, we have established a novel human cell line for therapeutic applications. We predict that HAT cells will provide a new and improved option for future biomanufacturing of gene therapy products.

## Materials and methods

### Ethics statement

This study was approved by the Ethics and Research Committees of the National Center for Child Health and Development (approval number: 2020-015) and the Manufacturing Technology Association of Biologics (approval number: 2019_001) and was conducted under the Ethical Guidelines for Life Science and Medical Research Involving Human Subjects by the Government of Japan. Written informed consent was obtained from all subjects before cesarean section.

### Isolation of amniotic epithelial cells

Human placentas were donated by healthy puerperae after scheduled cesarean deliveries. All donors were negative for HIV, HBV, HCV, HTLV-1, and *Treponema pallidum* on prenatal testing. hAECs were prepared as previously reported.^45^ Briefly, the amnion was mechanically peeled from the chorion and washed several times with saline (Otsuka Pharmaceutical Factory, Tokushima, Japan) containing 10 U/mL penicillin-streptomycin (Gibco, Billings, MT) and 2.5 μg/mL amphotericin B (Gibco). The amnion was then cut into small pieces and sterilized with 0.25% povidone-iodine (Toho Yakuhin, Tokyo, Japan). After washing with phosphate-buffered saline (PBS(−), Wako, Osaka, Japan) containing 10 U/mL penicillin-streptomycin and 2.5 μg/mL amphotericin B, the amnion pieces were transferred to PBS supplemented with 10 U/mL penicillin-streptomycin. To isolate hAECs, the amnion pieces were digested with trypsin-EDTA (0.05%) (Gibco) for 10 min, followed by two successive incubations with fresh trypsin-EDTA (0.05%) for 40 min at 37°C. Two volumes of hAEC culture medium (CTS KnockOut DMEM/F-12 [Gibco] supplemented with 10% [v/v] heat-inactivated fetal bovine serum [FBS] [Sigma-Aldrich, Burlington, MA], 100 U/mL Antibiotic-Antimycotic [Gibco], 2 mM GlutaMAX [Gibco], and 22 ng/mL human epidermal growth factor [Gibco]) were added to the cell suspensions from the second and third digestions. The essential supplements described earlier were always added to the culture medium, unless otherwise indicated. Single cells were released by repeated pipetting up and down and were resuspended and filtered through a 100 μm cell strainer (pluriSelect Life Science, Leipzig, Germany) to remove debris. After centrifugation at 200 × *g* for 10 min at 4°C, cell number and cell viability were determined using a Vi-CELL XR cell viability analyzer (Beckman Coulter, Brea, CA). hAECs were plated on 10-cm culture dishes at a viable cell density ranging from 5 × 10^6^ to 1 × 10^7^ cells/dish and maintained at 37°C in 5% CO_2_ and 5% O_2_.

### Establishment of immortalized HAT cells

A fragment consisting of bases 1–4,344 of the human AdV5 genome containing E1 regions was synthesized and cloned into the backbone vector pUCFa (FASMAC, Atsugi, Japan). The E1-encoding regions were PCR-amplified and purified using a column-based kit. DNA preparation was performed by FASMAC. The resultant double-stranded DNA product was named GT004 and was transfected into hAECs. Briefly, hAECs at 80%–90% confluency were dissociated using CTS TrypLE Select Enzyme (Gibco), and 2 × 10^6^ cells were plated on 6-cm culture dishes. The next day, the medium was removed and replaced with fresh hAEC medium without Antibiotic-Antimycotic. Several methods were used to transfect hAECs with 2.5 μg GT004, including Lipofectamine LTX (Lipo-LTX) with PLUS reagent (Invitrogen, Carlsbad, CA), Lipofectamine 2000 CD (Invitrogen). and ExPERT ATx (MaxCyte, Rockville, MD) according to the manufacturers’ instructions. At 6 h post-transfection, the medium was removed and replaced with fresh hAEC medium. The transfected cells were transferred to HAT culture medium (BenchStable DMEM/F-12 [Gibco] containing 10% FBS and 100 U/mL Antibiotic-Antimycotic [Gibco]) through a two-step process as described below. At 2 days post-transfection, the cells were detached, split 1:5 into 10-cm culture dishes with a 2:8 mixture of hAEC medium and HAT medium, and switched to normoxia conditions (20% O_2_). After culture for an additional 4 days, cells were transferred to full HAT culture conditions, and the medium was changed every 3–4 days. Approximately 30 days post-transfection, emerged transformed colonies were picked into 48-well plates using a cloning cylinder (Sigma-Aldrich) and cultured to confluency. Cells were passaged repeatedly to colonize 10-cm culture dishes for further development and assessment.

### Cell culture and transfection of adherent cells

HEK293 (CRL-1573) cells were purchased from the ATCC (Manassas, VA). HEK293 and HAT cells were maintained in BenchStable DMEM/F-12 containing 10% FBS and 50 μg/mL Gentamicin (Gibco) at 37°C in 5% CO_2_. To assess cell proliferation, 2.2 × 10^6^ cells were seeded on 10-cm culture dishes in triplicate. Cell number and cell viability were measured using a Vi-CELL XR analyzer (Beckman Coulter) at 1, 2, 3, and 4 days post-plating. To assess transfection efficiency, 3 × 10^6^ cells were seeded on 10-cm culture dishes and cultured overnight. The growth medium was replaced with fresh medium 1 h before transfection, and transfection was carried out using Lipo-LTX with PLUS reagent. Transfection complex mixtures contained 10 μg pAAV-ZsGreen1 (a ZsGreen1-expressing plasmid; Takara Bio, Kusatsu, Japan), 30 μL Lipo-LTX, and 30 μL PLUS regent in OptiPRO SFM (Gibco). Cell morphology and ZsGreen1 expression were observed under a BZ-X810 inverted fluorescence phase contrast microscopy (Keyence, Osaka, Japan). To quantify the percentage of ZsGreen1-positive cells, cells were harvested with CTS TrypLE Select Enzyme (Gibco), washed twice with Dulbecco PBS (DPBS) (Gibco), and fixed with 4% paraformaldehyde (Nacalai Tesque, Kyoto, Japan) in DPBS for 10 min at room temperature. Cells were then washed twice with DPBS and resuspended in 1 mL flow sytometry staining buffer (eBioscience, San Diego, CA). ZsGreen1 expression was analyzed using the SH800S cell sorter (Sony, Tokyo, Japan) and Cell Sorter Software (Sony).

### Serum-free suspension adaptation and single-cell cloning of HAT A-2 cells

After several passages of cells with a stable status (cells proliferating as expected with viability >95%) in 10-cm culture dishes containing HAT medium, adherent HAT A-2 cells were adapted to serum-free suspension culture through a four-step direct adaptation protocol. No antibiotics or antimycotics were used during and after the adaptation process. In brief, cells were first transferred from HAT medium to HE100 medium (Gmep, Kurume, Japan) containing 2 mM GlutaMAX. Once well adapted to this HE100 serum-free condition, HAT A-2 cells were switched to HE200 medium (Gmep) supplemented with 2 mM GlutaMAX and transferred to a 125-mL Erlenmeyer shake flask (Corning) and shaken at 120 rpm in a Lab-Therm LT-XC incubator shaker (Kuhner Shaker, Basel, Switzerland) with 8% CO_2_ and 85% humidity. After stabilization of proliferation and viability, HAT A-2 cells were then transferred to HE300AZ medium (Gmep) and finally to HE400AZ medium (Gmep), viral production medium (Gibco), or Expi293 expression medium (Gibco). Each cell inoculum was added to the fresh medium in conditioned medium to decrease stress. After adaptation, HAT A-2 cells were maintained in HE400AZ medium for growth and transfection unless otherwise specified. To obtain single cell-derived clones, a single-cell sorting and cloning technique using a SH800S cell sorter (Sony) was used in validated single cell mode. Clones were screened and selected in terms of proliferation capacity and AAV productivity. To assess cell proliferation, suspension HAT cells and VPCs 2.0 cells purchased from Gibco were seeded at a density of 0.3 × 10^6^ cells/mL in a 125-mL shake flask in triplicate. Cell number and cell viability were measured using a Vi-CELL XR analyzer (Beckman Coulter) at 2, 3, 4, and 5 days post-seeding.

### Short tandem repeat profiling

Genomic DNA was extracted from each sample using a DNeasy Blood & Tissue Kit (QIAGEN, Venlo, the Netherlands). DNA was amplified by multiplex PCR using the GenePrint 24 system (Promega, Madison, WI). The amplified products were analyzed by capillary electrophoresis using a 3730xl DNA analyzer (Applied Biosystems, Waltham, MA). Twenty-three short tandem repeat marker loci and the sex-specific marker Amelogenin were used to authenticate and determine the genetic identity of the HAT A-2 cells by comparison with the ATCC, DSMZ, and JCRB databases. Short tandem repeat analysis was performed by FASMAC.

### Karyotype analysis and identification of the E1 gene insertion site by FISH and multicolor FISH

FISH and multicolor FISH were performed by Chromosome Science Labo Inc. (Sapporo, Japan). In brief, log-phase adherent HAT A-2 and HAT A-12 cells were treated with 300 μg/mL thymidine overnight. After wash-out of thymidine, cells were cultured for 5 h, and then 0.02 μg/mL colcemid was added and culture continued for 1 h. Cells were harvested and resuspended in a hypotonic solution of 0.075 M KCl for 20 min and fixed with methanol-acetic acid (3:1) for 10 min. The fixed cells were hybridized with the 24XCyte human multicolor FISH probe (Metasystem, Reggio Emilia, Italy) and the Cy3-labeled AdV5 E1 DNA probe synthesized using the nick translation method. FISH probe signals were detected and analyzed using a CW-4000 cytogenetic workstation (Leica Microsystems, Wetzlar, Germany).

### Safety and identity testing

Safety and identity testing of HAT A2C1 cells was systematically conducted following the ICH Q5A guidelines.^46^ Assays included sterility testing by direct inoculation, adventitious agent testing using next-generation sequencing and transmission electron microscopy, mycoplasma detection via real-time PCR, and reverse transcriptase quantification by quantitative product-enhanced reverse transcriptase assays. *In vitro* assays were performed to assess potential viral contamination. Qualitative PCR assays were also used to detect human, bovine/porcine viruses, and viral DNA. Identity testing, including short tandem repeat assays, karyotype analysis, and CO1 barcode assays, was conducted in parallel. All assays were performed by BioReliance (Glasgow, UK).

### Western blotting

Rabbit polyclonal antibodies against E1B 19K and 55K proteins were generated with synthetic peptides corresponding to Q163–E176 of 19K and T483–D496 of 55K^47^ as antigens (Eurofins Genomics, Tokyo, Japan). Whole-cell lysates were prepared with RIPA lysis buffer (Thermo Scientific, Waltham, MA) containing cOmplete protease inhibitor cocktail (Roche, Basel, Switzerland). Protein concentrations were determined using the Quick Start Bradford protein assay (Bio-Rad, Hercules, CA) and bovine serum albumin (Bio-Rad) as standard. Samples were boiled at 95°C or heated at 42°C (for 55 K detection) for 5 min in Laemmli sample buffer (Bio-Rad) containing 2.5% 2-mercaptoethanol (Sigma-Aldrich). Equal amounts of proteins were resolved by sodium dodecyl sulfate-polyacrylamide gel electrophoresis on 12% gels and transferred onto 0.2 μm nitrocellulose membranes (Bio-Rad). The membranes were blocked in 5% skim milk (Wako, Osaka, Japan) in Tris-buffered saline containing 0.05% Tween 20 (Santa Cruz, Santa Cruz, CA) for 1 h at room temperature and were then probed with primary antibodies overnight at 4°C. Membranes were then incubated with horseradish peroxidase-conjugated secondary antibodies for 1 h at room temperature. The immunoreactive signals were developed using the Novex ECL chemiluminescent substrate reagent kit (Invitrogen) and imaged using an Amersham Imager 600 (GE Healthcare Life Sciences, Chicago, IL). Blots were probed with the following primary antibodies: E1A (M58) (1:500) (BD Pharmingen, San Diego, CA), E1B 19K (1:1,000), E1B 55K (1:100), GAPDH (14C10) (1:1,000) (Cell Signaling, Danvers, MA). The secondary antibodies were horseradish peroxidase-conjugated anti-mouse-immunoglobulin G (IgG) (A16078) (1:10,000) (Invitrogen) and anti-rabbit-IgG (ab205718) (1:10,000) (Abcam, Cambridge, United Kingdom).

### RT-qPCR

Total RNA was extracted from 1 × 10^6^ cultured cells using an RNeasy Plus Mini Kit (QIAGEN), and 1 μg of total RNA was reverse transcribed to cDNA using a QuantiTect reverse-transcription kit (QIAGEN). After 10-fold dilution, 1 μL of each cDNA sample was subjected to PCR amplification. qPCR mixtures were prepared with PowerUp SYBR Green master mix (Applied Biosystems). Quantitative reverse-transcription PCR (RT-qPCR) was performed using the QuantStudio 6 Pro real-time PCR instrument (Applied Biosystems) with the following program recommended by the manufacturer: 50°C for 2 min, 95°C for 2 min, 40 cycles of 3-step PCR of 95°C for 15 s, 58°C for 15 s, and 72°C for 1 min, followed by a melting curve stage of 95°C for 15 s, 60°C for 1 min, and 95°C for 15 s. The primer sets used were 5′-GGAGCAAGTTGCAAAGCATTG-3′ and 5′-TCCCACGACGTAGTCCATGTT-3′ for *hTERT*,^48^ and 5′-CTGGAACGGTGAAGGTGACA-3′ and 5′-AAGGGACTTCCTGTAACAACGCA-3′ for β-actin.^49^ Every sample was run in triplicate, and data were analyzed by the ΔΔCt method.

### TRAP assay

Telomerase activity was determined by TRAP assays using a PCR-based TRAPeze telomerase detection kit (Millipore, Burlington, MA). Briefly, cell lysates were prepared by incubating 1 × 10^6^ cells with 200 μL CHAPS lysis buffer. The lysates were incubated on ice for 30 min and centrifuged at 12,000 × *g* for 20 min at 4°C. Supernatant aliquots were quick-frozen in liquid nitrogen. Telomerase activity was inactivated by heat treatment at 85°C for 10 min. Sample aliquots containing 2 × 10^3^, 5 × 10^2^, and 1 × 10^2^ HAT A-2 and HEK293 cells and 2 × 10^3^ hAECs and heat-treated HAT A-2 and HEK293 cells were subjected to PCR amplification. CHAPS lysis buffer and TSR8 provided by the kit were included as negative and positive controls, respectively. The following program was used for PCR: telomerase extension at 30°C for 30 min, followed by 95°C for 2 min, then 30 cycles of 3-step PCR of denaturation at 94°C for 15 s, annealing at 59°C for 30 s, and extension at 72°C for 1 min on a C1000 Touch thermal cycler (Bio-Rad). The PCR products were separated by 5%–20% native polyacrylamide gel electrophoresis (ATTO, Tokyo, Japan), and the gel was stained with SYBR Gold (Invitrogen) and imaged using an Amersham Imager 600 (GE Healthcare Life Sciences).

### Flow cytometry

Cells were harvested using CTS TrypLE Select Enzyme (Gibco), resuspended in DPBS, and fixed in 80% cold methanol for 5 min at −20°C. After two DPBS wash steps, the cells were permeabilized with 0.1% Tween 20 (Wako) for 20 min at room temperature, followed by blocking with 10% normal goat serum (Abcam) for 10 min at room temperature. Antibody staining was performed using pan-CK (C11) Alexa Fluor 488 (2 μg per 1 × 10^6^ cells) (sc-8018 AF488; Santa Cruz) or normal mouse IgG1 Alexa Fluor 488 (sc-3890; Santa Cruz) as an isotype control for 30 min at room temperature, protected from light. Cells were then washed with DPBS, resuspended in 0.5 mL flow cytometry staining buffer, and analyzed on an SH800S cell sorter with Cell Sorter Software (Sony).

### AAV vector production using adherent cells

Cells were seeded at 6 × 10^6^ cells/10-cm culture dish in complete medium without antibiotics and cultured overnight. Transfection was performed using Lipo-LTX with PLUS Reagent. Lipo-LTX was used at a Lipo:DNA ratio of 1:1. The plasmid DNA was transfected at a ratio of 1:1:1 (21.6:21.6:21.6 μg per 10-cm dish) for pRC and pHelper from the AAVpro helper-free system (Takara Bio) and pAAV-ZsGreen1 as the gene of interest. The estimated amount of plasmid DNA used was approximately 5.5 μg/1 × 10^6^ cells. To harvest AAV particles, the transfected cells were lysed in AAV-MAX lysis buffer (Gibco) at 72 h post-transfection according to the manufacturer’s instructions with some modifications. Briefly, 1 mL of AAV-MAX lysis buffer was added at a 1:10 dilution directly to the 10-cm dish, and the cells were incubated at 37°C for 15 min. The supernatant and detached cells were collected into a 50 mL centrifugation tube, and the lysis reaction was continued by incubation at 37°C for 1 h at 250 rpm on a WB-101SRC orbital shaker (WAKENBTECH, Kyoto, Japan). The lysate was centrifuged at 3,000 × *g* for 10 min, and 800 μL of the supernatant was collected into a 1.5 mL microcentrifuge tube and centrifuged again at 10,000 × *g* for 10 min. The supernatant containing crude AAV lysate was transferred to a new 1.5 mL microcentrifuge tube and stored at −80°C until further analysis.

### AAV vector production using suspension cells

On the day of transfection, HAT A-2, HAT A2C1, and VPCs 2.0 cells were counted and diluted to a viable cell density of 2 × 10^6^ cells/mL. Twenty-milliliter cultures in 125-mL shake flasks were prepared for transfection. Various off-the-shelf transfection reagents were screened, as described in Figure S5B. The standard triple plasmid transfection was performed using FectoVIR-AAV (Polyplus, Illkirch, France) in HE400AZ medium. The transfection cocktails, corresponding to 20% of the volume of the cell cultures after dilution, included pRC and pHelper from the AAVpro helper-free system and pAAV-ZsGreen1 as the gene of interest at a ratio of 1:1:1. For AAV8 vector production, an additional experiment was performed that used pPLUS AAV-Helper plasmid in place of pHelper to assess the impact of different helper plasmids. Plasmid DNA and FectoVIR-AAV were applied at doses of 1 μg/1 × 10^6^ cells and 1 μL/1 × 10^6^ cells, respectively. The cocktail was mixed thoroughly by gentle inversion. After incubation for 1 h at room temperature, the cocktail was added to the cell culture. Seventy-two hours after transfection, the cells were lysed using AAV-MAX lysis buffer at 1:10 dilution (typically, 100 μL buffer per 900 μL of harvested culture), as described in the “AAV vector production using adherent cells” section, except that only one centrifugation was performed at 3,000 × *g* for 5 min.

### Large-scale AAV vector production using an orbital shaken bioreactor

Four days before transfection, cells were counted and diluted to a viable cell density of 0.4 × 10^6^ cells/mL in an SB10-X single-use orbital shaken bioreactor (Kuhner, Basel, Switzerland). On the day of transfection, cells were counted and diluted to a viable cell density of 2 × 10^6^ cells/mL in 3 L. The transfection and lysis procedure were performed as described earlier in the “AAV vector production using suspension cells” section, with proportional scale-up. pPLUS Helper-AAV plasmid was used in the large-scale experiment.

### ddPCR

ddPCR was performed according to the manufacturer’s instructions for the QX200 ddPCR system (Bio-Rad). Crude AAV lysates were treated with 10 U DNase I (Takara Bio) at 37°C for 30 min and then cooled to 4°C. Samples were then serially diluted in the range of 1:1,000 to 1:4,000 in a poly(A) solution (10 mM Tris-HCl, pH 8.0, 0.1 mM EDTA, 100 μg/mL poly(A), 0.01% Pluronic F-68), depending on the original sample titers. The diluted samples were incubated at 95°C for 10 min and then cooled to 4°C. Each 22 μL sample mixture consisted of 5.5 μL template, 250 nM probes, and 900 nM primers. Droplets were generated by an automated droplet generator (Bio-Rad), and PCR was run with the following program: 95°C for 10 min, followed by 40 cycles of 94°C for 30 s and 55°C for 1 min, and a final step of 98°C for 10 min on a C1000 Touch thermal cycler (Bio-Rad).

For quantification of AAV genomic titer, crude lysates were used. Reaction mixtures were prepared with ddPCR Supermix for Probes (No dUTP) (Bio-Rad), ddPCR Expert Design Assay (AAV-ITR2 [HEX] [Bio-Rad]), and ddPCR Expert Design Assay (CMV promoter [FAM] [Bio-Rad]). Both probes yielded comparable results across samples, and the figures show the ITR probe-derived titers. For analysis of genome regions packaged in AAV particles, affinity-purified AAV samples were used. Reaction mixtures were prepared using ddPCR Copy-Number Variation Assays (FAM) (Bio-Rad) targeting various regions across the AAV vector genome, spanning from the plasmid backbone, through the AAV ITRs, enhancer, transgene, and poly(A) signal (see Table S1).^50^ To minimize interference from ITR secondary structures, 5 U of SmaI restriction enzyme (Takara Bio) were added to the PCR reaction mixtures. The PCR results were analyzed using QuantaSoft Software (v.1.7) (Bio-Rad). For AAV empty-to-full ratio quantification, ddPCR was performed using the Vericheck ddPCR Empty-Full Capsid Kit (Bio-Rad) according to the manufacturer’s instructions. Briefly, DNase-treated AAV crude lysates were diluted 1:10 (AAV2) or 1:100 (AAV8) and first incubated with serotype-specific antibody, followed by a ligation step. ddPCR reaction mixture contained 4.4 μL of stopped ligation reaction as template, ddPCR Supermix for Probes, ddPCR Capsid Detection Assay (FAM), and ddPCR ITR2 Assay (HEX). The results were analyzed using QX Manager Software (v.2.3) (Bio-Rad). The percentage of full capsids was calculated as the ratio of genome to capsid concentration.

### AEX chromatography purification

Crude AAV lysates were clarified by 0.2 μm surfactant-free cellulose acetate syringe filtration (Corning). The clarified lysates were then subjected to affinity capture chromatography using a POROS GoPure AAVX pre-packed column (Thermo Scientific) on an Äkta avant 25 system (GE Healthcare Life Sciences). The affinity purification column was equilibrated with five column volumes of equilibration buffer (20 mM Tris, 200 mM NaCl, pH 7.8). After sample loading, the column was washed with ten column volumes of equilibration buffer. The AAV capsids were eluted with five column volumes of elution buffer (50 mM glycine, pH 2.7) and neutralized by the addition of 10% v/v of neutralization buffer (1 M Tris, pH 9.0). To separate empty and full capsids, the affinity-purified samples were subjected to AEX using Capto Q AEX resin (Cytiva, Marlborough, MA) and eluted using a gradient of mobile phase A containing 20 mM Bis-Tris Propane in 2 mM MgCl_2_ (pH 9.0) and mobile phase B containing mobile phase A supplemented with 250 mM sodium acetate. The separation was conducted using a linear salt gradient from 0% to 75% mobile phase B over thirty column volumes, during which an isocratic hold at 24% mobile phase B (corresponding to approximately 6 mS/cm conductivity) was incorporated for fifteen column volumes to improve the separation of empty and full capsids. To increase the purity of full capsids, fractions of full particles were harvested and re-applied to a second AEX purification run under the same conditions. The runs were monitored at UV absorbance of 260 and 280 nm.

### Mass photometry

Mass photometry measurements were performed using a SamuxMP mass photometer (Refeyn, Oxford, UK) and AcquireMP software (2025 R1.2) (Refeyn) on a sample well cassette and sample carrier slide purchased from Refeyn. MassFerence P2 calibrant (Refeyn), a standard protein of 3640 kDa, was used as a reference for mass calibration prior to each measurement. To start the measurement, 10 μL PBS (Gibco) was loaded into the well of the cassette. After automatic focus, 10 μL of affinity-purified AAV sample, pre-diluted in PBS to approximately 10^11^ virus particles per milliliter, was added and mixed by pipetting with the buffer drop. Each measurement was recorded as a 1-min video, and each sample was measured in triplicate. The results were analyzed using DiscoverMP software (v.2025 R1) (Refeyn), and the peaks of the mass histograms were applied to a Gaussian fit to quantify the percentage of empty and full capsids.

### Residual hcDNA quantification

Residual hcDNA was extracted from 100 μL affinity-purified AAV samples using the High Pure Viral Nucleic Acid Kit (Roche) according to the manufacturer’s instructions, except that the Proteinase K digestion was extended to 1 h to fully release encapsidated DNA. An aliquot of the extracted DNA (10 μL out of 50 μL) was used as the template to detect residual hcDNA using a qPCR-based ResDNASEQ quantitative human DNA kit (Applied Biosystems) following the manufacturer’s protocol. Genomic DNA extracted from HAT A2C1 cells and VPCs 2.0 cells was included as positive controls. qPCR was performed on a QuantStudio 6 Pro real-time PCR instrument (Applied Biosystems) with the following program: 95°C for 10 min, followed by 40 cycles of 95°C for 15 s and 60°C for 1 min.

### LC-FLR/MS

LC-FLR/MSanalysis was performed using a BioAccord LC-MS system (Waters, Milford, MA) incorporated with an ACQUITY UPLC I-Class PLUS system coupled with an ACQUITY UPLC FLR detector and an ACQUITY RDa time-of-flight mass spectrometry detector based on a previously reported method with minor modifications.^30^ To analyze AAV capsid proteins, affinity-purified AAV samples were denatured with 10% (v/v) acetic acid (Fujifilm Wako, Osaka, Japan) for 15 min. Ten microliters of the treated samples was injected onto an ACQUITY UPLC BEH C_4_ column (1.7 μm, 300Å, 2.1 × 100 mm; Waters) at a flow rate of 0.2 mL/min and column temperature of 80°C. Mobile phase A and mobile phase B were 0.1% difluoroacetic acid (Waters) in LC-MS-grade water and acetonitrile, respectively. The LC gradient was modified as follows: initially 20% mobile phase B, increased to 32% over 1.5 min, followed by a linear gradient from 32% to 36% over 24 min. Mobile phase B was then increased to 80% over 30 min, held at 80% until 32.25 min, and re-equilibrated to 20% from 33 to 45 min. Mass spectrometric detection was conducted using the RDa mass detector in positive electrospray ionization mode, following previously reported settings^30^ with the scan rate adjusted to 5 Hz. Raw MS spectra were processed using the MaxEnt1 algorithm within the waters_connect informatics platform to obtain deconvoluted masses of intact viral proteins. For viral protein stoichiometry analysis, FLR detection was performed at a 5 Hz scan rate, with excitation at 280 nm and emission at 350 nm.

### AAV transduction assay

HEK293 cells were seeded at a density of 3 × 10^5^ cells/well in a 24-well plate containing BenchStable DMEM/F-12 (Gibco) supplemented with 10% FBS and incubated overnight. The culture medium was changed to DMEM/F-12 medium containing 2% FBS and diluted affinity-purified AAV2-ZsGreen1 vectors produced by HAT A2C1 or VPCs 2.0 cells at MOIs ranging from 5 × 10^2^ to 1 × 10^4^. At 96 h post-infection, cells were stained with Hoechst 33342 (Invitrogen) and imaged for ZsGreen1 expression using BZ-X810 microscopy. Transduction was also performed using full particle-enriched samples from AEX purification at MOIs ranging from 1 × 10^2^ to 5 × 10^3^. The percentage of ZsGreen1-positive cells was quantified using a Countess 3 FL automated cell counter (Invitrogen) and was calculated from biological triplicates for affinity-purified samples and from technical triplicates of one AEX-purified sample transduction experiment.

### Statistical analysis

All data are presented as the mean ± standard deviation from three independent experiments, unless otherwise stated. For certain assays, technical replicates were performed as indicated in the figure legends. Assessment of statistically significant differences was performed using the unpaired, two-tailed Welch’s t test (unpaired, unequal variance) for pairwise comparisons. A *p* value < 0.05 was considered statistically significant.

## Data and code availability

The data supporting the findings of this study are available from the corresponding authors upon reasonable request.

## Acknowledgments

This work was supported by a grant-in-aid for Research and Development of Core Technologies for Gene and Cell Therapy from the Japan Agency for Medical Research and Development (AMED) (grant number: JP18ae0201001 and JP24se0123004). We thank Jeremy Allen, PhD, from Edanz (https://jp.edanz.com/ac) for editing a draft of this manuscript.

## Author contributions

Y.H., T.H., and K.N. conceptualized the study and supervised the entire project; Y.H., Y.-H.C., R.A., and K.N. designed the experiments; Y.H., Y.-H.C., A.Y., R.A., R.M., M. Kinoshita, M.M., and M. Kubota conducted the experiments; Y.H., K.A., M. Kubota, T.H., and K.N. provided access to human resected material; Y.-H.C. prepared the figures and wrote the initial and final drafts of the manuscript; Y.H. and K.N. provided critical advice on refining the manuscript. All authors reviewed and approved the final version of the manuscript.

## Declaration of interests

Y.H., Y.-H.C., A.Y., R.A., R.M., M. Kinoshita, M. Kubota., and T.H. are employees of Chitose Laboratory Inc.

## Supplemental information

Document S1. Figures S1–S10 and Tables S1–S4

Document S2. Article plus supplemental information
