# Supplementary material for: Establishment of a novel human amniotic epithelial-derived cell line, HAT, for high-yield AAV vector production
Source: Mol Ther Methods Clin Dev. 2025 Sep 12;33(4):101594. doi: 10.1016/j.omtm.2025.101594 (PMC12495061; doi:10.1016/j.omtm.2025.101594)
Supplement: Document S1. Figures S1–S10 and Tables S1–S4 [file mmc1.pdf]

OMTM, Volume *33*

Supplemental information

Establishment of a novel human amniotic epithelial-derived cell line, HAT, for high-yield AAV vector production

Yugo Hirai, Yu-Hsin Chang, Arisa Yamamoto, Ryo Asahina, Rena Moromizato, Mawo Kinoshita, Kazuko Aizawa, Manami Miyai, Michi Kubota, Takayuki Horiuchi, and Kazuaki Nakamura

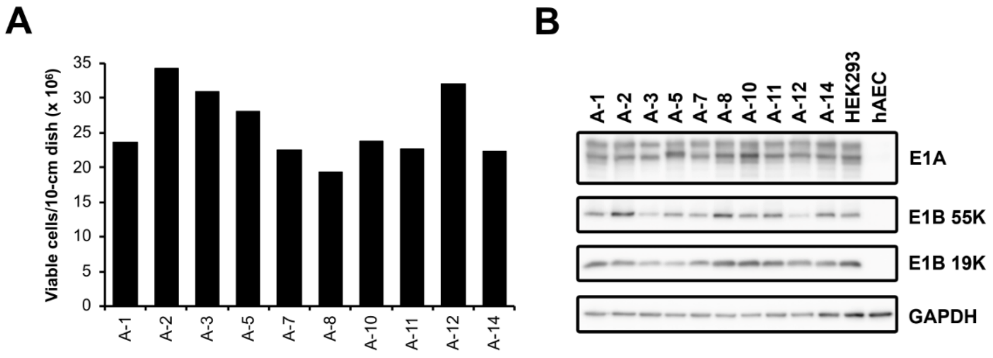

Figure S1. Candidate HAT cell lines. (A) Cells were seeded at a density of 1×10^6 cells in a 10-cm dish. The culture medium was changed on day 5 after plating. Cells were subcultured on day 7, and viable cell numbers were counted. The total viable cell number per 10-cm dish is shown. (B) Protein levels of AdV5 E1A and E1B were examined by western blotting. Major E1A isoforms were detected. GAPDH served as a loading control. Results were obtained from a one-time screen performed during the early stage of cell line development.

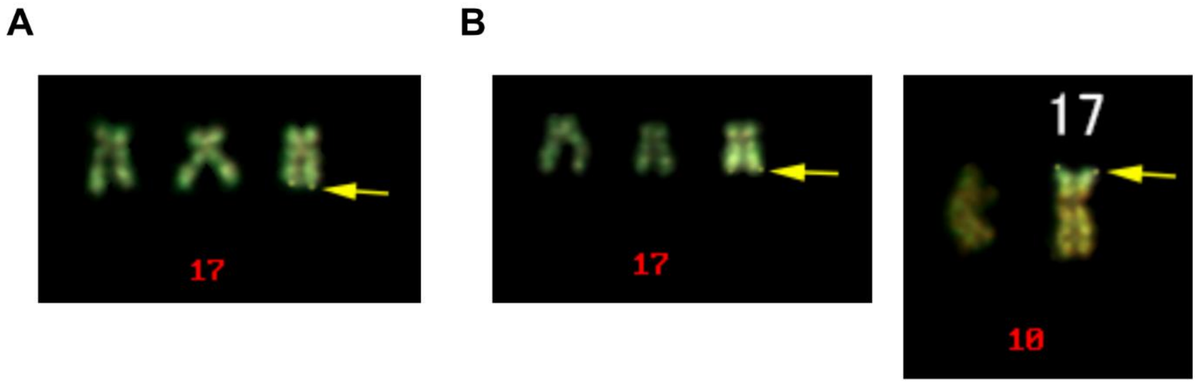

Figure S2. Insertion site of the AdV5 E1 gene in HAT A-12 cells. E1 integration sites were analyzed by multicolor-FISH and FISH using E1-specific DNA probes (indicated by the arrows). Two patterns were identified. (A) FISH signals were observed on the telomeric region of the long arm of chromosome 17. 17: chromosome 17. (B) FISH signals were detected on both chromosome 17 (left) and chromosome 10 (right), within a translocated fragment from the telomeric region of the long arm of chromosome 17. 17: chromosome 17, 10: chromosome 10.

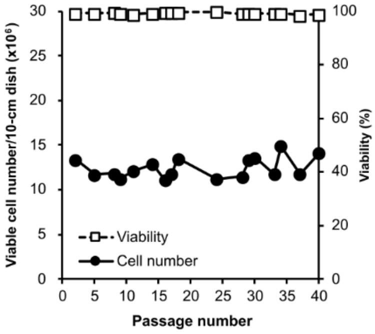

Figure S3. Long-term growth of adherent HAT A-2 cells. For routine maintenance, HAT A-2 cells were seeded at a density of 1×10^6 cells in a 10-cm dish. Cells were subcultured every 3 or 4 days, and viable cell numbers were counted. The total viable cell number per 10-cm dish obtained on day 3 of routine passages is plotted to represent long-term growth trends. Data collected on passage day 4 are excluded to ensure data comparability and consistency.

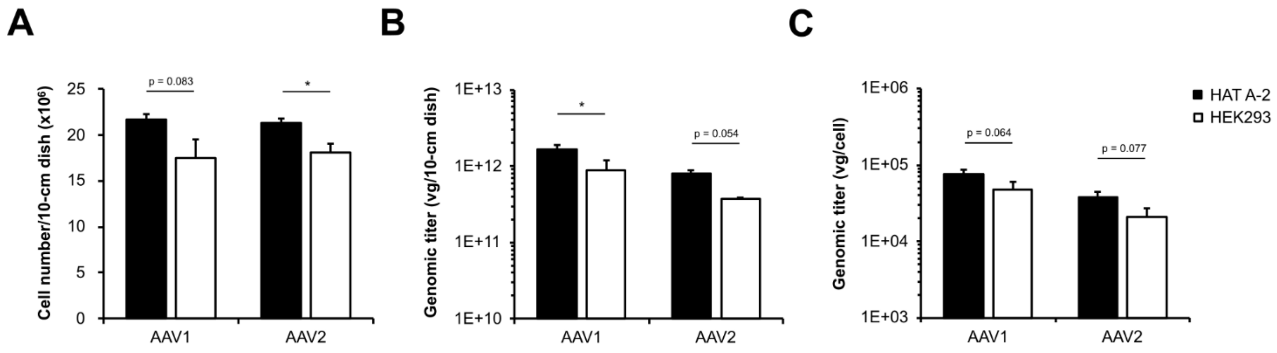

Figure S4. AAV production metrics of adherent HAT A-2 cells. AAV1 and AAV2 were produced by a triple plasmid transfection method in 10-cm dishes. (A) Cell numbers were counted at 72 hours post-transfection before sample harvesting. (B) Total AAV yield per 10-cm dish was measured by ddPCR. (C) Cell-specific productivity was calculated as total AAV yield per dish divided by the corresponding cell number. *$p < 0.05$. P values between 0.05 and 0.1 are shown to indicate near-significant trends.

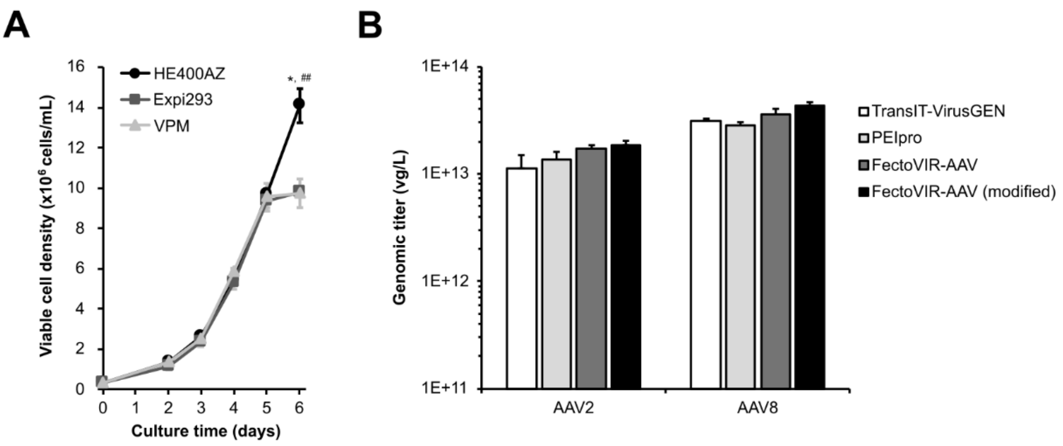

Figure S5. Evaluation of suspension HAT A-2 cells for cell growth and AAV productivity across various culture media and transfection reagents. (A) HAT A-2 cells were adapted to several commercially available culture media. To assess cell growth, cells were seeded at a density of 0.3 × 10^6 cells/mL in a 125-mL shake flask containing 30 mL medium in triplicate. Cell numbers were counted daily for 6 days. Statistical comparisons were performed against HE400AZ conditions. *$p < 0.05$ vs. Expi293; ##$p < 0.01$ vs. VPM. Expi293: Expi293 Expression Medium; VPM: Viral Production Medium. (B) To screen for compatibility and assess AAV productivity, AAV2 and AAV8 vectors were produced using triple plasmid transfection according to the recommended protocols of each manufacturer. Briefly, the plasmid DNA was transfected at a ratio of 1:1:1 for pRC, pHelper from the AAVpro Helper Free System, and pAAV-ZsGreen1 as the gene of interest. Plasmid DNA and transfection reagent were mixed at 1 µg DNA per 1 µl reagent. The transfection cocktails were prepared at 10% of the culture volume for TransIT-VirusGEN (Mirus Bio, Madison, WI) and PEIpro (Polyplus). FectorVIR-AAV was tested under both manufacturer-recommended (5%) and modified (20%) complexation volumes. The complexation time was 15 minutes for TransIT-VirusGEN and PEIpro, 30 minutes for the manufacturer-recommended conditions of FectoVIR-AAV, and 60 minutes for the modified conditions. Genomic titers were measured by ddPCR.

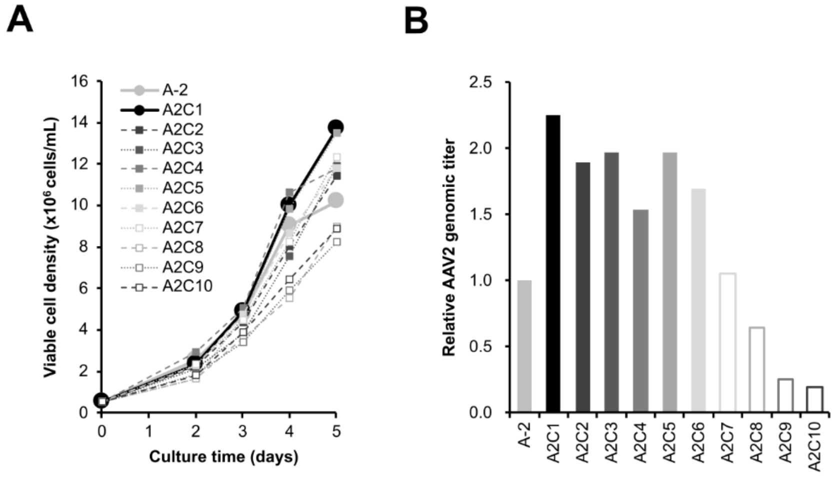

Figure S6. Evaluation of 10 representative single-cell clones derived from HAT A-2 cells. (A) Cells were seeded at 0.5 × 10^6 cells/mL in a 500-mL shake flask containing 120 mL medium. Cell numbers were counted daily for 5 days. (B) AAV2 vectors were produced using a triple plasmid transfection method. Genomic titers were measured by qPCR using AAVpro Titration Kit (for Real Time PCR) Ver.2 (Takara Bio). Fold changes in volumetric productivity produced from candidate clones were calculated relative to HAT A-2 cells. Results from 10 representative clones, selected from the final 60 candidates, are shown from a single evaluation experiment.

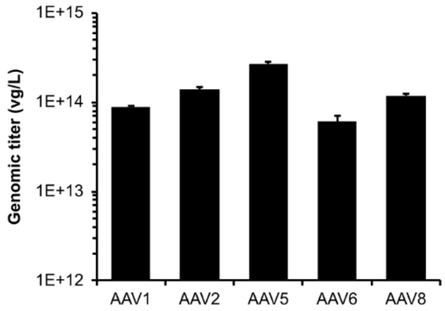

Figure S7. AAV productivity in HAT A2C1 cells after plasmid ratio adjustment. AAV vectors were produced using the standard triple plasmid transfection protocol described in the 'AAV vector production using suspension cells' section, except that the pRC:pHelper:pAAV-ZsGreen1 ratio was adjusted to 1.72:3.16:1. Genomic titers were measured by ddPCR. A representative result from a transfection experiment performed in technical triplicates is shown.

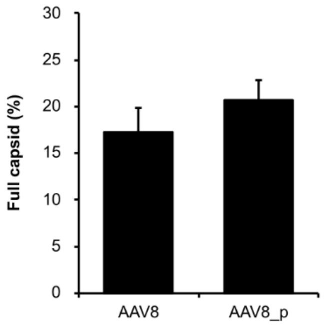

Figure S8. Comparison of full AAV8 capsids under two helper plasmid conditions in HAT A2C1 cells. AAV8 vectors were produced using either the pHelper plasmid from the AAVpro Helper Free System (AAV8) or the pPLUS AAV-Helper plasmid (AAV8_p). These experiments were performed in parallel with the shake flask experiments shown in Figure 7. The percentage of full capsids was determined as the genome-to-capsid titer ratio measured using the Vericheck ddPCR Empty-Full Capsid Kit. The pPLUS condition was selected for subsequent large-scale production because of the higher proportion of full capsids.

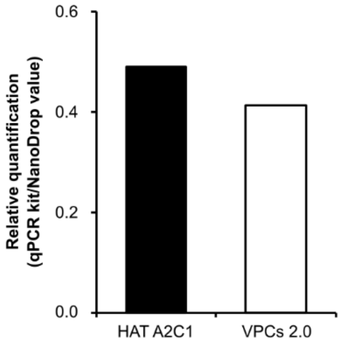

Figure S9. Genomic DNA quantification to verify the reliability of hcDNA detection. Genomic DNA (gDNA) was extracted using a DNeasy Blood & Tissue Kit (QIAGEN) according to the manufacturer's instructions and quantified using a qPCR-based ResDNASEQ Quantitative Human DNA Kit and a NanoDrop One/One spectrophotometer (Thermo Scientific, Waltham, MA). Values represent the ratio of genomic DNA concentration measured by the qPCR kit to that measured by NanoDrop. Data are from a single experiment.

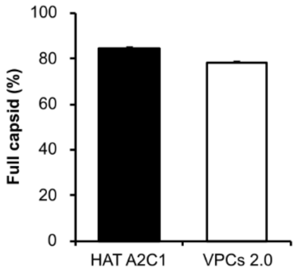

Figure S10. Enrichment of AAV2 full capsids following two consecutive rounds of AEX chromatography. AAV2 vectors were produced in 1-L shake flasks using the standard triple transfection method described in the 'AAV vector production using suspension cells' section, with proportional scale-up. The percentage of full capsids was determined based on the genome-to-capsid titer ratio, measured using a Vericheck ddPCR Empty-Full Capsid Kit. Data represent a single experiment. Error bars indicate standard deviation of ddPCR technical triplicates.

Table S1. List of primer/probe mixes for ddPCR copy number assays used for whole genome region mapping.

Assay ID	Mapped region
dCNS114927258	5′ plasmid backbone – 5′ ITR
dCNS186312283	5′ ITR – CMV enhancer
dCNS683934997	ZsGreen1
dCNS568970821	ZsGreen1
dCNS953120020	hGH poly(A) – 3′ ITR
dCNS787498924	3′ plasmid backbone

Table S2. Assignment of AAV2 capsid proteins

	Identity	AA sequence	Observed mass (Da)	Theoretical mass (Da)
HAT A2C1	VP1	2(Ac)–735	81856.0	81856
	VP2	139–735	66488.3	66488
	VP3	204(Ac)–735	59973.0	59974
VPCs 2.0	VP1	2(Ac)–735	81855.0	81856
	VP2	139–735	66487.7	66488
	VP3	204(Ac)–735	59973.0	59974

The observed mass was calculated as the mean of three biological replicates.

Table S3. Assignment of AAV8 capsid proteins

	Identity	AA sequence	Observed mass (Da)	Theoretical mass (Da)
HAT A2C1 3-L	VP1	2(Ac)–738	81667	81667
	VP2	139–738	66517	66519
	VP3	205(Ac)–738	59804	59805
	VP3 clip	205(Ac)–659	50593	50593
HAT A2C1 125-mL	VP1	2(Ac)–738	81667.0	81667
	VP2	139–738	66519.0	66519
	VP3	205(Ac)–738	59804.0	59805
	VP3 clip	205(Ac)–659	50593.0	50593
VPCs 2.0 125-mL	VP1	2(Ac)–738	81666.7	81667
	VP2	139–738	66518.0	66519
	VP3	205(Ac)–738	59804.0	59805
	VP3 clip	205(Ac)–659	50593.0	50593

The observed mass was calculated as the mean of three biological replicates for the shake flask conditions and from a single experiment for the bioreactor conditions.

Table S4. Safety and identity testing of HAT A2C1 cells.

Assay	Result
Sterility testing	Pass
Adventitious agent by next-generation sequencing	No viral sequences of significance were detected
Transmission electron microscopy	No extraneous agents observed
Mycoplasma detection	Negative to a sensitivity of 10 cfu/5E5 cells
Quantitative product-enhanced reverse transcriptase assay	Negative for the presence of retroviral reverse transcriptase
In vitro assay for viral contaminants	Pass
Qualitative PCR for human, bovine/porcine viruses, and viral DNA	Negative to detection limit
Karyotype analysis	Consistent with a cell line of human origin
CO1 barcode assay	Homology with human
